# Chemistry of Hydrogen Sulfide—Pathological and Physiological Functions in Mammalian Cells

**DOI:** 10.3390/cells12232684

**Published:** 2023-11-22

**Authors:** Celia María Curieses Andrés, José Manuel Pérez de la Lastra, Celia Andrés Juan, Francisco J. Plou, Eduardo Pérez-Lebeña

**Affiliations:** 1 Hospital Clínico Universitario of Valladolid, Avenida de Ramón y Cajal, 3, 47003 Valladolid, Spain; cmcurieses@gmail.com; 2 Institute of Natural Products and Agrobiology, CSIC-Spanish Research Council, Avda. Astrofísico Fco. Sánchez, 3, 38206 La Laguna, Spain; 3 Cinquima Institute and Department of Organic Chemistry, Faculty of Sciences, Valladolid University, Paseo de Belén, 7, 47011 Valladolid, Spain; celia.andres.juan@uva.es; 4 Institute of Catalysis and Petrochemistry, CSIC-Spanish Research Council, 28049 Madrid, Spain; fplou@icp.csic.es; 5 Sistemas de Biotecnología y Recursos Naturales, 47625 Valladolid, Spain; info@glize.eu

**Keywords:** hydrogen sulfide, chemistry, gasotransmitter, physiology

## Abstract

Hydrogen sulfide (H_2_S) was recognized as a gaseous signaling molecule, similar to nitric oxide (-NO) and carbon monoxide (CO). The aim of this review is to provide an overview of the formation of hydrogen sulfide (H_2_S) in the human body. H_2_S is synthesized by enzymatic processes involving cysteine and several enzymes, including cystathionine-β-synthase (CBS), cystathionine-γ-lyase (CSE), cysteine aminotransferase (CAT), 3-mercaptopyruvate sulfurtransferase (3MST) and D-amino acid oxidase (DAO). The physiological and pathological effects of hydrogen sulfide (H_2_S) on various systems in the human body have led to extensive research efforts to develop appropriate methods to deliver H_2_S under conditions that mimic physiological settings and respond to various stimuli. These functions span a wide spectrum, ranging from effects on the endocrine system and cellular lifespan to protection of liver and kidney function. The exact physiological and hazardous thresholds of hydrogen sulfide (H_2_S) in the human body are currently not well understood and need to be researched in depth. This article provides an overview of the physiological significance of H_2_S in the human body. It highlights the various sources of H_2_S production in different situations and examines existing techniques for detecting this gas.

## 1. Introduction

Hydrogen sulfide (H_2_S) [1] is currently considered a physiological modulator in mammals. It can be formed in mammalian cells and is an important gasotransmitter that plays important physiological and pathophysiological roles. H_2_S is the most recently discovered endogenous gasotransmitter and, like ^•^NO and CO, crosses cell membranes without a specific transporter [2]. H_2_S can be generated by various processes and can undergo chemical reactions with metal centers and oxidized thiol compounds, especially asymmetric disulfides (R_1_SSR_2_). Initially considered a toxic molecule, it is now recognized that mammalian cells are endowed with enzymatic pathways for H_2_S production. The identification of H_2_S as a gasotransmitter has led to a renewed interest during the last two decades, focusing on (i) creating chemical tools for its physiological detection, (ii) determining its signaling functions in various organs and systems in the plant and animal kingdoms and (iii) exploiting its biological signaling capacity for therapeutic benefits.

**Figure 1:**
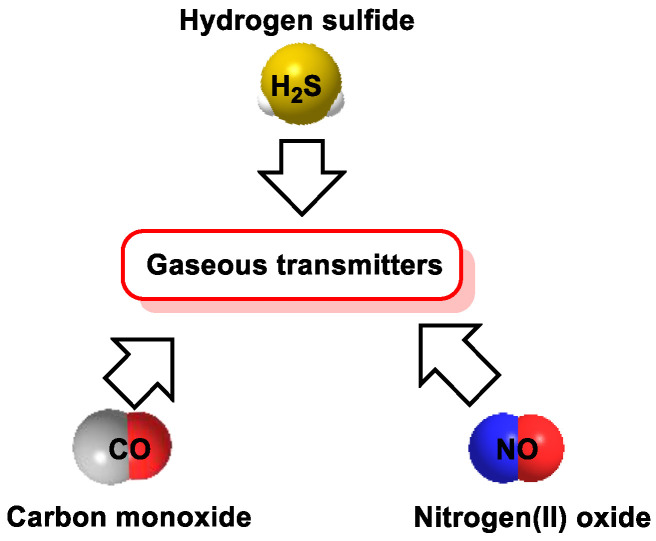
^•^NO, CO and H_2_S are gaseous signaling molecules involved in biological functions.

In the late 20th century, H_2_S was discovered to be an endogenously produced gas in tissues. It is synthesized from the amino acid L-cysteine, D-cysteine, homocysteine, cystathione and 3-mercaptopyruvate by a series of enzymes whose expression and molecular regulation in various tissues have been characterized by several groups. It is an endogenous mediator of inflammation [3] and a potential cytoprotective compound [4]. At that time, H_2_S was found to be generated enzymatically, and it was included in the family of gaseous transmitters (also due to its chemical properties). ^•^NO, CO and H_2_S are part of the group of gasotransmitters (Figure 1).

The expression of H_2_S-catabolic catabolic enzymes varies in numerous physiological systems, including the endocrine, neuronal, cardiovascular, respiratory, immunological, reproductive, renal, hepatic and gastrointestinal systems. Various enzymes play a role in modulating the activities of different systems by regulating the generation of H_2_S. Pathological conditions such as atherosclerosis, hypertension, heart failure, cirrhosis, inflammation, asthma, sepsis, diabetes, erectile dysfunction and neurodegenerative diseases arise because of alterations in H_2_S metabolism [5,6,7].

During the First World War, the British army used H_2_S as a chemical weapon. However, its suitability as a combat gas was compromised by its flammability and unique odor, which could serve as an indicator of the gas’s location to the enemy [8]. Today it is known to act as a second messenger and is associated with important functions at the level of blood vessel walls [9]. The properties attributed to it are antioxidant, anti-atherogenic, anti-apoptosis and anti-inflammatory properties, as well as anti-proliferative, neuromodulatory and cytoprotective properties [10].

## 2. H_2_S Chemistry

H_2_S is a gas at room temperature, heavier than air, colorless with a characteristic rotten egg odor and toxic [11,12,13]. Until the mid-1990s, it was considered mainly toxic to humans and harmful to the environment. The substance is moderately soluble in water, with a solubility of 2.77 volumes in 1 volume of water at a temperature of 20 °C. Moreover, it can be effectively eliminated from an aqueous solution by complete dissolution during boiling.

**Figure 2:**
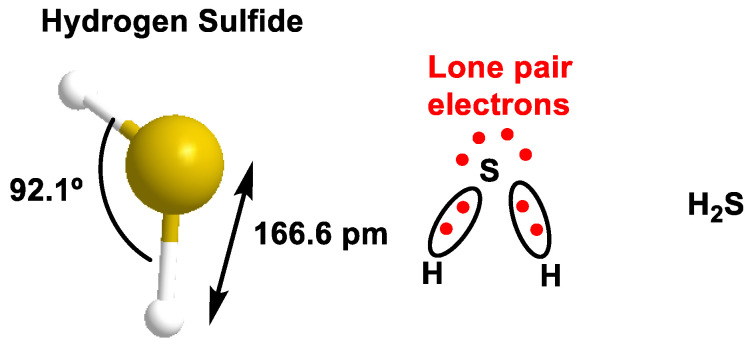
H_2_S molecule structural formula, molecular geometry, angle and Lewis structure.

A sulfur atom is larger than an oxygen atom (covalent radius 105 vs. 66) and has a comparatively lower electronegativity value of 2.5 on the Pauling scale, while oxygen (O) has a higher electronegativity value of 3.5. In addition, sulfur is characterized by a higher degree of polarizability. Consequently, the dipole moment of H_2_S is comparatively smaller than that of water, being 0.97 D and 1.85 D, respectively, and intermolecular interactions are weaker. It has a similar structure to water, but since sulfur has lower electronegativity values than oxygen (S = 2.5 and O = 3.5, according to the Pauling scale), H_2_S is less polar than water [14]. Compared to water, this difference has an impact on the inability to form hydrogen bridges. H_2_S can diffuse freely through the hydrophobic core of biological membranes [15]. The sulfur in H_2_S has the electronic configuration 1s^2^, 2s^2^ 2p^6^, 3s^2^ and 3p^6^ and borrows an electron from each hydrogen to complete its valence shell (Figure 2).

H_2_S is considered a toxic substance when inhaled in its pure form or even when diluted in a ratio of 1 part gas to 200 parts air. Bird species are very sensitive to H_2_S, rendering them susceptible to mortality even at a dilute concentration of 1 part per 1500 in atmospheric air. H_2_S has significant inhibitory effects on specific enzymes and processes related to oxidative phosphorylation, ultimately leading to cell suffocation. Most people perceive hydrogen sulfide through the sense of smell when the concentration is above 5 parts per billion (ppb). Concentrations of 20 to 50 parts per million (ppm) have been found to cause irritation to both the eyes and the respiratory system.

Short-term inhalation of 100 to 250 parts per million (ppm) can cause adverse effects such as incoordination and cognitive and motor impairment. At concentrations of about 150 to 200 parts per million (ppm), individuals may suffer from olfactory fatigue or anosmia, resulting in an inability to detect the characteristic odor of H_2_S. Inhalation of a concentration of 500 parts per million (ppm) for a period of 30 min may result in the development of pulmonary edema and pneumonia.

Concentrations greater than 600 parts per million (ppm) have the potential to cause death within a short 30 min period, primarily due to paralysis of the respiratory system. A concentration of 800 ppm is considered immediately fatal to humans. H_2_S has a comparatively high solubility in water, 110 millimoles per atmosphere at standard room temperature [16,17].

H_2_S is the smallest and simplest inorganic biologically relevant thiol with the maximal reduced state of sulfur atom (−2) [18]. H_2_S has only oxidizing properties and does not have the ability to act as an oxidant. H_2_S is a reducing species, with a reduction potential of −280 mV (pH 7.0 vs. standard hydrogen electrode) for the two-electron redox pair HS^−^/S^0^ and −230 mV for H_2_S/S^0^, close to the values for the glutathione/glutathione disulfide (GSSG/GSH) and cystine/cysteine redox couples [19]. The first evidence for the physiological role of H_2_S was its detection in brain tissues of different species [20].

H_2_S serves as a stimulator of electron transport in mammalian mitochondria by acting as an electron donor (the electron acceptor is oxidized CoQ), involving sulfur-quinone oxidoreductase (SQR) and leading to the production of GSSH-glutathione persulfide or thiosulfate and reduced CoQ [21].

The H_2_S-derived effect on electron transport processes is a double-edged sword: a low amount can stimulate respiration, while a higher dose will lead to complete inhibition of respiration by inhibiting the heme-containing proteins. It is still questionable whether H_2_S has any impact on the electron transport chain via enrichment of the reduced CoQ pool, particularly under normoxic (in vivo) or supra-normoxic conditions (21% O_2_, in cell incubators [22,23].

## 3. Pathways of H_2_S Production

### 3.1. Environmental (Anthropogenic and Non-Anthropogenic) Sources

Non-anthropogenic environmental sources of H_2_S are wetlands, volcanic eruptions and emissions, geothermal activity, thermal upwellings and sulfide-metabolizing bacteria. Anthropogenic emissions of H_2_S are paper factories, tanneries, mining, oil refineries, combustion and sewage dumps.

### 3.2. Endogenous Sources of H_2_S in Mammalian Cells

In 1996, it was shown that cystathionine-β-synthase can produce H_2_S in the brain and that H_2_S facilitates the induction of hippocampal long-term potentiation by enhancing NMDA receptor activity [24]. In 1997, another H_2_S-producing enzyme, cystathionine-γ-lyase, was found to be expressed in the thoracic aorta, portal vein and ileum, and it was found that H_2_S relaxes these tissues [25]. Based on these findings, H_2_S may be a neuromodulator as well as a smooth muscle relaxant, in addition to functioning as a signaling molecule and as a cytoprotectant [4]. H_2_S protects neurons from oxidative stress by restoring depleted glutathione levels. A third H_2_S-producing enzyme, 3-mercaptopyruvate sulfurtransferase (3MST), is expressed in neurons, the vascular endothelium [26] and the mitochondria of almost all cells [27]. In addition to restoring glutathione levels, the H_2_S produced by 3MST, which is mainly located in the mitochondria, reduces ROS generated in these organelles [24].

The biosynthetic pathways leading to H_2_S production rely on the activity of three enzymes: cystathionine-γ-lyase (CSE), cystathionine-β-synthase (CBS) and 3MST, which synthesize H_2_S in vivo (CSE from L-cysteine, CBS from homocysteine and 3MST from mercaptopyruvate) [28]. CBS is the main H_2_S generator in the brain, while CSE generates H_2_S in peripheral tissues, and both enzymes are present in both the central nervous system and peripheral tissues. The third enzyme involved in H_2_S production, 3MST, also functions in the brain [29].

Selenium-Binding Protein 1 (SELENBP1) supports H_2_S biosynthesis and adipogenesis. The cellular regulatory functions of SELENBP1 are not fully understood [30]. Subsequently, another pathway was documented in which H_2_S is produced using D-cysteine as a substrate in conjunction with the enzymes D-amino acid oxidase and 3MST [31,32]. The gut microbiota also produces H_2_S, which has a significant impact on the physiological processes of both the host organism and the microbial community. H_2_S has the potential to be released in a living organism. There are two major sources that have the ability to release H_2_S. The first is the labile acid pool, which includes proteins with iron–sulfur groups. The second source is the sulfane sulfur pool, which functions in the presence of reducing agents [33]. In addition to an immediate release of H_2_S from the producing enzymes, H_2_S can be stored as sulfane sulfur (any sulfur atom bound to another sulfur atom and an ionizable hydrogen).

There are at least two mechanisms for H_2_S release [33]:

(i) H_2_S is released immediately after its production by enzymes;
(ii) H_2_S is stored and released in response to a physiological signal.

And there are two forms of sulfur deposition in cells [34,35]:

1. Acidic conditions release H_2_S from acid-labile sulfur. Acid-labile sulfur is mainly sulfur atoms in iron–sulfur complexes, which play a key role in a wide range of redox reactions in respiratory chain enzymes in mitochondria. The critical pH below which H_2_S is released from labile sulfur to acid is 5.4 [36]. Because the mitochondrial pH is between 7 and 8, which is higher than the critical pH, acid-labile sulfur may not release H_2_S under physiological conditions.
2. Another form of storage is called sulfane sulfur, which is localized in the cytoplasm, releasing H_2_S under reducing conditions [37]. This includes compounds such as polysulfides, thiosulfates, polyethionates, thiosulfonates, bisorganyl-polysulfanes or monoarylthiosulfonates and elemental sulfur. Sulfane sulfur compounds such as polysulfides release H_2_S under reducing conditions, suggesting that the cellular redox state is important in regulating their bioavailability [38]. The activity of reducing substances increases under alkaline conditions, and H_2_S can be released from sulfane sulfur when intracellular conditions become alkaline. In addition, free H_2_S can be incorporated into proteins as sulfane sulfur, where its divalent sulfur form binds only elemental sulfur, persulfides and polysulfides [39].

H_2_S is produced in almost all mammalian cell types. In humans, there are at least two sources of H_2_S generation: (i) enzymatic synthesis in tissues and (ii) metabolism by bacteria in the digestive tract [40]. Erythrocytes are also capable of reducing elemental sulfur to HS^−^ in a non-enzymatic manner [41].

Non-enzymatic H_2_S production occurs via glucose, glutathione, organic and inorganic polysulfides and elemental sulfur. The reduction of elemental sulfur using NADPH or the oxidation of glucose in the phosphogluconate pathway also produces H_2_S in a non-enzymatic form [41].

**Figure 3:**
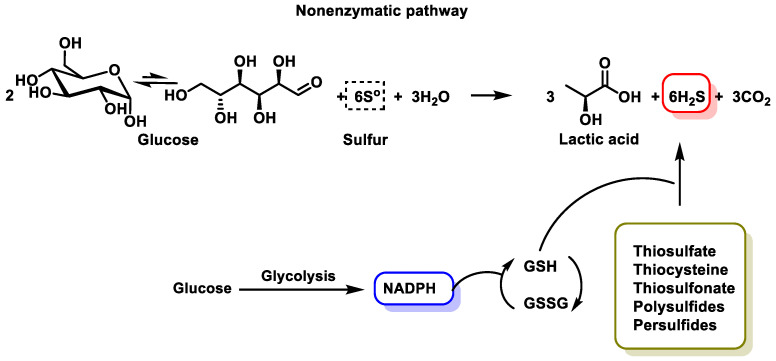
Various non-enzymatic routes of H_2_S synthesis. In the presence of reducing equivalents such as NADPH and NADH, reactive sulfur species in persulfides, thiosulfate and polysulfides are reduced into H_2_S and other metabolites. GSH is glutathione and GSSG is glutathione disulfide.

The non-enzymatic form, although less important, comes from the reduction of elemental sulfur to H_2_S using reductants that come from glucose oxidation, such as lactate, or others such as nicotinamide adenine dinucleotide hydrogen (NADH), nicotinamide adenine dinucleotide phosphate (NADPH) and glutathione (Figure 3).

The non-enzymatic pathway involves the production of NADPH as a by-product of glycolysis to drive the reduction of glutathione disulfide (GSSG) to liberate H_2_S from sulfane sulfur compounds [42].

Non-enzymatic H_2_S production in eukaryotes requires various forms of sulfane such as thiosulfate, thiocysteine, polysulfides, persulfides, thiosulfonate and elemental sulfur to release H_2_S in the presence of reactive molecules [43]. The oxidation of glucose in glycolysis generates reducing equivalents, such as NADPH, which are used for the reduction of glutathione disulfide to glutathione. In addition, this process leads to the release of H_2_S from the above-mentioned sulfur-containing compounds [41].

**Figure 4:**
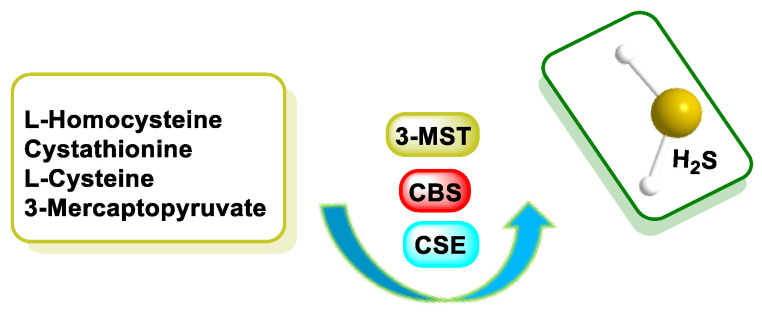
The biosynthesis of H_2_S mammalian cells primarily involves cystathionine β-synthase (CBS), cystathionine-γ-lyase (CSE) and 3-mercaptopyruvate sulfurtransferase (3MST).

Among the enzymes involved in sulfide synthesis, cystathionine-β-synthase, cystathionine-γ-lyase and mercaptopyruvatesulfur transferase are part of the transsulfuration pathway, the latter involved in cysteine catabolism (Figure 4).

**Figure 5:**
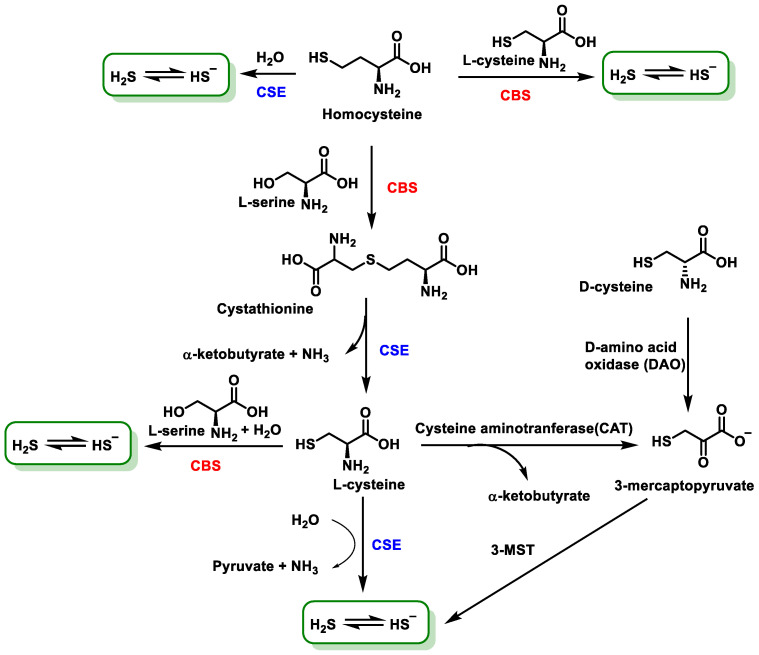
Main route of H_2_S synthesis mediated by the enzymes CBS, CSE and 3MST.

The enzymatic pathway involves the production of H_2_S from L-cysteine, which is an amino acid synthesized by the organism. In mammals, the enzymatic synthesis of H_2_S involves two pyridoxal-5′-phosphate-dependent enzymes located in the cytosol: cystathionine β-synthase (CBS) and cystathionine γ-lyase (CSE) [44]. A third H_2_S-producing enzyme, 3MST, is expressed in neurons and the vascular endothelium. In addition, 3MST and cysteine aminotransferase (CAT) contribute to H_2_S biosynthesis [45]. The third enzyme mentioned, 3MST, unlike the other two which are only in the cytosol, is found in both cytosol and mitochondria [46] and catalyzes the anaerobic pathway of cysteine conversion together with cysteine aminotransferase, transporting sulfide ions to thiol (-SH) groups and forming H_2_S. This enzyme is localized in the kidney, liver, heart, lungs, thymus, testis, brain and erythrocytes [41,47] (Figure 5).

Cystathionine-β-synthase (CBS) is the predominant enzyme in the production of H_2_S in the brain and nervous system; is present in liver, kidney, ileum, uterus, placenta and pancreatic islets [46]; belongs to the family of carbon-oxygen-lyases; and is a hydrolase as it catalyzes the cleavage of a C-O bond in serine, with the loss of a water molecule. Human CBS is a homopolymer and uses pyridoxal-5′-phosphate (PLP) as a cofactor, and each monomer contains a non-catalytic heme cofactor [44]. CBS can produce H_2_S by three distinct pathways:

(1) Hydrolysis of cysteine to form serine and H_2_S.
(2) Condensation of cysteine and homocysteine to generate cystathionine and H_2_S.
(3) Condensation of two cysteine molecules to form lanthionine and H_2_S [48].

Cystathionine-γ-lyase (CSE) is capable of producing H_2_S via both cysteine (70%) and homocysteine (30%) and is expressed in liver, kidney, ileum, uterus, brain, pancreatic islets and placenta, as well as being characteristic of the vascular system and its smooth muscle [49]. CSE is a PLP-using enzyme, and its catalytic mechanism is very similar to that of CBS. It is a homotetramer [50].

H_2_S can also be generated from L-methionine via transsulfuration [51]. Homocysteine, a risk factor for cardiovascular and neurocognitive diseases, is converted to H_2_S, a cardiovascular and neuronal protector, via the transsulfuration pathway or the release of a persulfide (RS-SH) by thiol–disulfide exchange [51].

**Figure 6:**
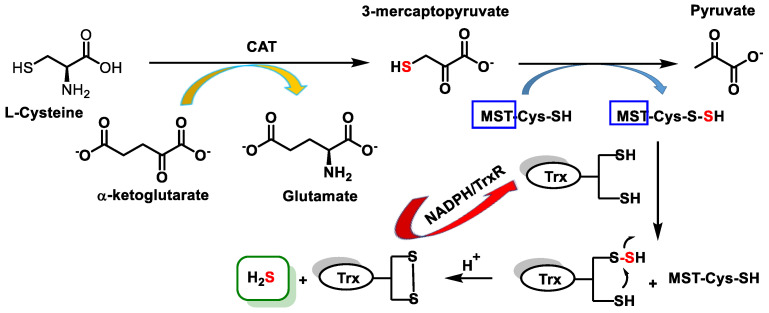
H_2_S synthesis via the 3MST/CAT pathway.

Mercaptopyruvatesulfurtransferase (3MST) belongs to the family of sulfurtransferases, which catalyze the transfer of a sulfur from a sulfur donor to a thiophilic acceptor by the formation of a protein cysteine persulfur intermediate [52]. The sulfur atom donor is 3-mercaptopyruvate, and the kinetically favored acceptor under physiological conditions is thioredoxin [53] (Figure 6).

The enzyme known as cysteine aminotransferase (CAT) facilitates the transamination process of cysteine and α-ketoglutarate, leading to the production of mercaptopyruvate and glutamate, respectively. Subsequently, another enzyme called mercaptopyruvate sulfurtransferase (MST) catalyzes the transfer of sulfur from 3-MP to a specific active cysteine site. This transfer leads to the formation of pyruvate and a persulfide compound bound to MST. The persulfide bound to MST then reacts with thioredoxin and eventually produces H_2_S [53].

**Figure 7:**
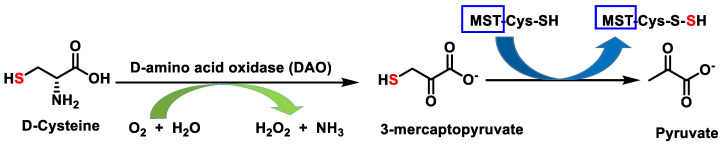
Formation of 3-mercaptopyruvate from D-cysteine.

A synthesis pathway has been described from D-cysteine, which by action of the enzyme D-amino acid oxidase (DAO) produces 3-mercaptopyruvate, which serves as a substrate for 3MST [54] (Figure 7).

In contrast to the L-cysteine system, the D-cysteine-dependent metabolic pathway functions mainly in the cerebellum and kidney, and it is approximately 80 times more efficient than CAT/3MST in H_2_S production in these tissues [32,55].

### 3.3. Microbial Synthesis of H_2_S

**Figure 8:**
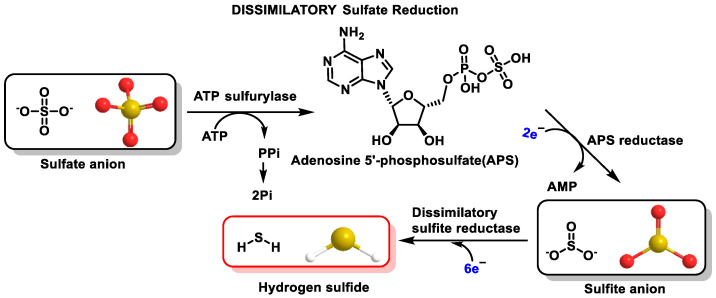
Dissimilatory sulfate reduction pathway.

Some intestinal bacteria use sulfate as a final acceptor in their respiratory chain and produce H_2_S, implying that there is an additional source of sulfide in this tissue, in addition to the basal production in the intestinal cells from CBS and CSE enzymes. This results in the organs of the digestive tract, mainly the intestinal epithelium, being exposed to high concentrations of the gas [56,57]. However, the amount of H_2_S produced by the microbiota is variable and may be related to diet and the type of microbiota present in the gut [58]. H_2_S has a major influence on digestive health and can be generated from the degradation of cysteine, as well as through the dissimilatory reduction of sulfate [59] (Figure 8).

Sulfate reduction, depending on the micro-organism, can take place in two ways: assimilatory and dissimilatory [60,61]. In dissimilatory sulfate reduction, H_2_S is produced, and in the assimilatory form, the terminal product is cysteine. The above pathways show clear differences, which are mainly characterized by the role of dissimilatory sulfate in energy production and the function of sulfate as an electron acceptor. In the process of assimilatory sulfate reduction, sulfate serves as a substrate for the production of amino acids.

Sulfate-reducing bacteria are responsible for generating 50% of the H_2_S found in colonocytes, while the other 50% corresponds to the action of CBS and CSE enzymes [62].

### 3.4. Production in Laboratory or Industrially

In ambient conditions, hydrogen gas (H_2_) and the element sulfur (S) show no reactivity. However, when temperatures above room temperature are reached, these elements initiate a chemical reaction, with the optimal temperature being 310 °C.

**Figure 9:**
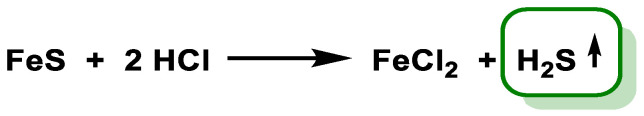
Mineral-acid-catalyzed H_2_S synthesis.

However, since this process is very slow, alternative methods of extraction are used, such as the one shown in the figure below. Metal sulfides, including iron sulfide, react with acids, such as hydrochloric acid, in a dilute solution (Figure 9).

In this way, H_2_S gas is obtained and, due to its toxicity, must be collected safely.

## 4. H_2_S Consumption Routes

**Figure 10:**
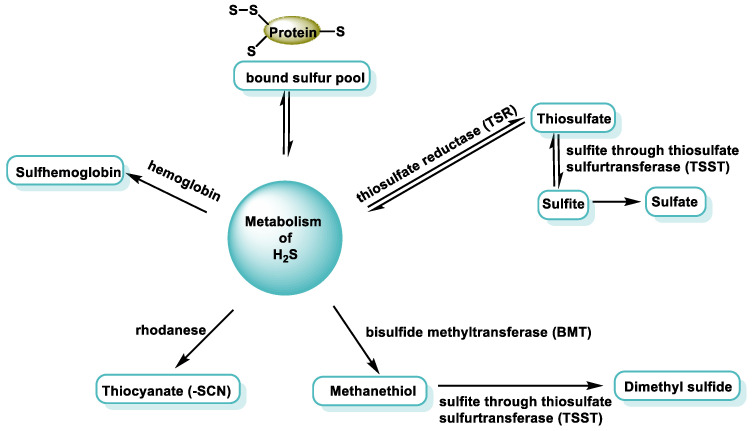
The metabolic processes involved in the breakdown and utilization of H_2_S. H_2_S is degraded by various enzymatic reactions. The enzymes rhodanese, bisulfide methyltransferase (BMT) and thiosulfate reductase (TSR) play critical roles in catalyzing the conversion of H_2_S to thiocyanate, methanethiol and thiosulfate, respectively. Oxidation of thiosulfate to sulfite can occur through the enzymatic action of thiosulfate sulfurtransferase (TSST), followed by further oxidation to sulfate. H_2_S reacts with hemoglobin, resulting in the formation of sulfohemoglobin. In addition, H_2_S combines with proteins present in the tissue, resulting in the formation of a bound sulfur pool.

There are five ways of eliminating H_2_S in the body (Figure 10).

It is important to control the concentration of H_2_S in mammalian tissues, which is maintained at nanomolar levels [63]. The concentration of H_2_S varies between different tissues, and the general agreement is that its concentration should be present in nanomolar concentration, as higher concentrations can inhibit metal-containing proteins. However, methods for the detection of H_2_S are currently being developed.

H_2_S only takes on an important signaling function at low concentrations. At higher concentrations, however, it has the potential to have a toxic effect by inhibiting cell respiration through the inhibition of cytochrome c oxidase (CcOX) [64]. To prevent toxicity, it is necessary to regulate the bioavailability of H_2_S [65].

### 4.1. Mitochondrial Pathway of Sulfur Oxidation

In the body, H_2_S is broken down by the mitochondrial enzyme system, currently designated as the “sulfuroxidising unit” or “sulfuroxidising pathway” [66], which transforms sulfur into sulfate and thiosulfate.

**Figure 11:**
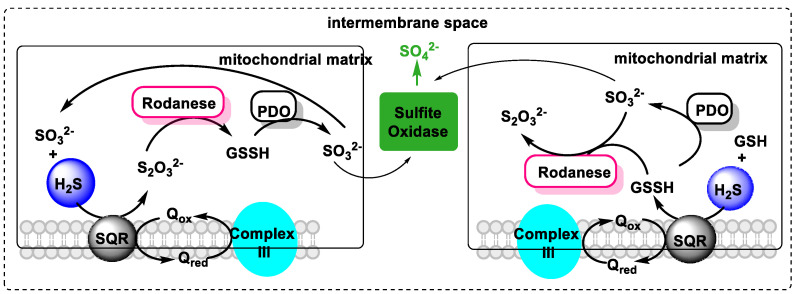
The two alternative modes for sulfide oxidation.

Four enzymes are involved in the oxidation pathway: (i) One is sulfur quinone oxidoreductase (SQR), which catalyzes the transfer of two electrons from sulfur to oxidized coenzyme Q. The sulfur is oxidized and transferred to an acceptor molecule (GSH or sulfite). (ii) Others are sulfur transferase (rhodanase) and (iii) oxidases such as persulfide dioxygenase (PDO) and sulfite oxidase (Figure 11). Sulfate and thiosulfate are the main products of the mammalian mitochondrial sulfide oxidation pathway and are safer than sulfide [14].

According to the current knowledge, SQR is the ancient enzyme (from algae and bacteria) initially used instead of Complex I to provide the electrons from H_2_S to the rest of the primitive ETC process, mostly consisting in its beginning of SQR and the primitive version of Complex IV and Complex V [67]. Mitochondria have the main intracellular machinery responsible for the uptake of H_2_S and its oxidation into inert metabolites: sulfite and sulfate [68].

Globins and other proteins, including catalase and superoxide dismutase, can serve as catalysts for the oxidation of H_2_S to polysulfide. This process involves the use of O_2_ or H_2_O_2_ as an electron acceptor [65,69,70,71,72].

### 4.2. Oxidation of Sulfides by Hemoproteins

Sulfur can react with metal centers through covalent bonding, redox interaction or coordination. This is described as another possible mechanism of sulfide detoxification in red blood cells. As there are no mitochondria in red blood cells, oxidation of sulfur by the route described above is not possible [73].

**Figure 12:**
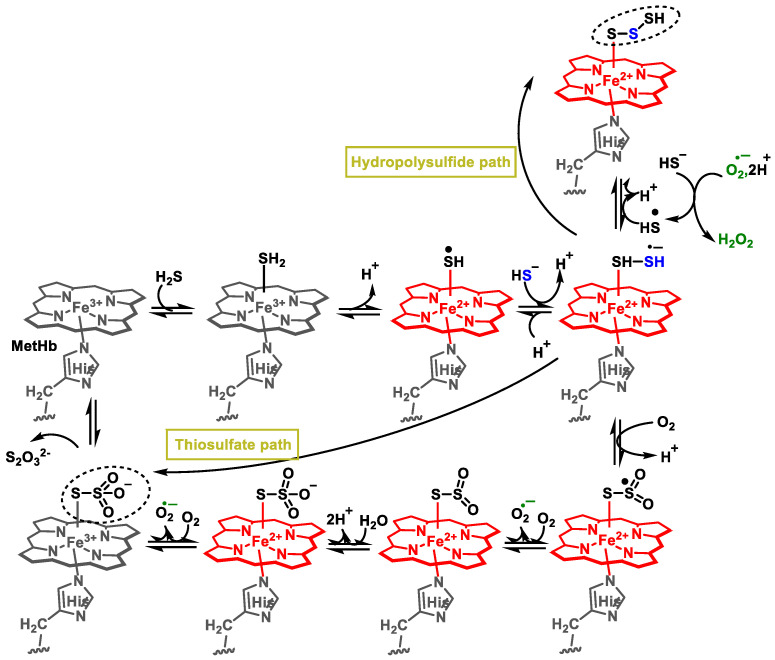
Reaction mechanism postulated for the oxidation of H_2_S by MetHb. Methemoglobin binds to H_2_S and oxidizes it to a mixture of thiosulfate and hydropolysulfides.

In methemoglobin, iron is present as Fe(III), which can react reversibly with H_2_S to form an intermediate MetHb-Fe(III)-SH_2_. H_2_S can dissociate and subsequently replenish iron heme through a reversible mechanism. Alternatively, it has the potential to convert ferrous heme into its ferrous counterpart. This redox process would be favored by the loss of a proton from H_2_S bound to the heme. Methehemoglobin binds to H_2_S, and the end result is oxidation to a mixture of thiosulfate and hydropolysulfides (Figure 12).

Like human Hb and Mb, there is a set of enzymes such as globins, lactoperoxidases and catalase that react with H_2_S to form sulfemo analog derivatives, where a sulfur atom covalently bonds to one of the pyrroles of the porphyrin ring [74].

## 5. H_2_S Reactivity

H_2_S has strong nucleophilic properties that allow it to react with electrophilic species, whereas sulfur is a good reductant and will react with oxidants. Sulfur has a variable oxidation state, which can range from −2 to +6, and, in its lowest oxidation state, −2, is a good reductant. This means that it can give up electrons to other atoms, reducing its oxidation state. The sulfur atom from H_2_S is also a good reductant because sulfur has an oxidation state of −2. When H_2_S reacts with an oxidant, the sulfur gives up electrons to the oxidant, reducing its oxidation state. It is, therefore, a good reductant acting as a general sulfur atom or as a sulfur atom from H_2_S.

**Figure 13:**
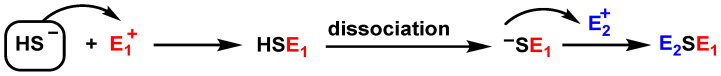
HS^−^ reaction with two different nucleophiles.

These properties have been used to make probes to determine the amount of H_2_S generated. As shown in Figure 13, sulfur can act as a double nucleophile, and this is an important difference from cysteine.

### 5.1. In Aqueous Solution

**Figure 14:**
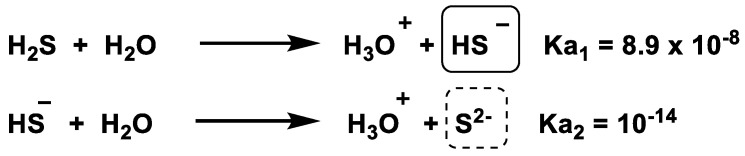
H_2_S dissociation equilibria.

H_2_S is a weak acid that possesses two ionizable protons [75] (Figure 14).

The limited ionization of the first proton can be derived from the initial ionization constant. The ionization of the second proton is minimal, but solutions of hydrogen sulfide contain a certain amount of the sulfur anion S^2−^ [76].

Under physiological conditions, at pH 7.4, the monoanionic species (HS^−^) will predominate by 71.5%, while the remaining 28.5% will be in the protonated form (H_2_S [65]; under these conditions, the concentration of the dianionic species (S_2_^−^) is considered negligible.

In the alkaline mitochondrial matrix, which is characterized by a pH of 8.0, about 92% of the sulfur species present are in the form of HS^−^, while the remaining 8% are due to H_2_S. HS^−^ is a prominent sulfur species that exerts a strong attraction to cell nuclei by binding to specific metal centers found in some molecules, such as the oxygen binding site of hemoglobin [76]. The enormous biological potential of H_2_S is attributed to this phenomenon. In the acidic environment of lysosomes, which is characterized by a pH of 4.7, most H_2_S is present in its H_2_S form, which accounts for over 99% of the compound. In this state, H_2_S has low polarity, allowing it to move freely, diffuse and accumulate in an aqueous or hydrophobic medium, such as biological membranes. About 40% of the sulfides present in the body are in the form of H_2_S, while the remaining portion is present as hydrogen sulfide anion (HS^−^). There is a tiny and potentially insignificant amount of S^2−^. The fundamental difference of H_2_S from the other gasotransmitters is its ability to dissociate in a solution.

### 5.2. Reaction with Oxygen

**Figure 15:**
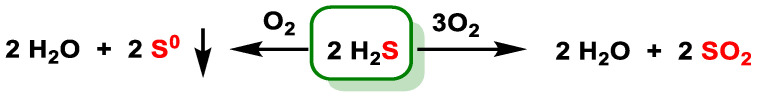
Reaction of H_2_S with oxygen.

H_2_S interacts with the oxygen present in the atmosphere, leading to the following chemical reactions (Figure 15).

### 5.3. Basic Chemical Reactivity of H_2_S

**Figure 16:**
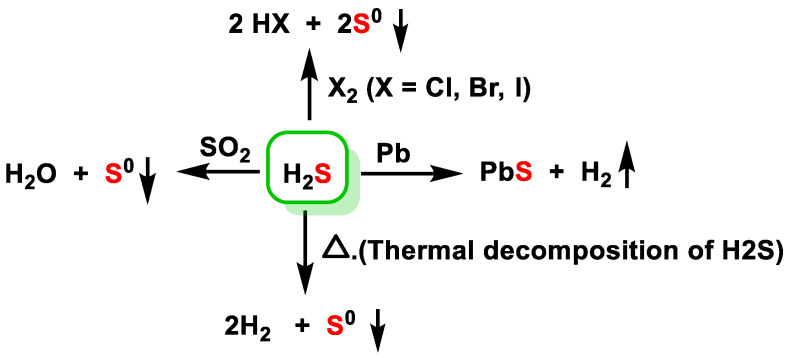
Chemical reactivity of H_2_S.

The basic chemical reactivity of H_2_S is summarized in Figure 16.

The formation of hydrogen halides and sulfur takes place in the presence of chlorine Cl_2_, bromine Br_2_ and iodine I_2_ [77]. H_2_S reacts with metals by forming metal sulfide [78]. H_2_S and SO_2_ are present in volcanic gases and react with each other to form solid sulfur [79]. H_2_S is not very stable; it decomposes easily upon heating [80].

### 5.4. Reaction with Disulfide

**Figure 17:**
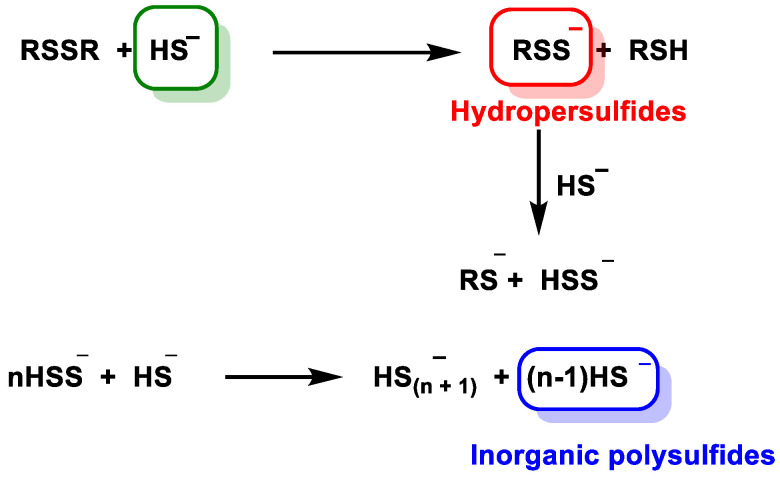
Reaction of disulfide reduction by HS^−^.

Kinetic studies showed that the reaction of disulfide reduction by HS^−^ occurs through multistep equilibria with the formation of disulfide, hydropersulfide and inorganic polysulfides [81]. The mechanism presented in the figure indicates that inorganic polysulfides (HS_n_^−^) are formed in parallel with hydrosulfides (RSS^−^) (Figure 17).

Inorganic polysulfides have been identified as potential intermediates in the transition pathway from sulfhydryl (SH) to persulfide (SSH) species and are thought to play a role in thiol-based redox signaling mechanisms.

## 6. Biological Functions of H_2_S

**Figure 18:**
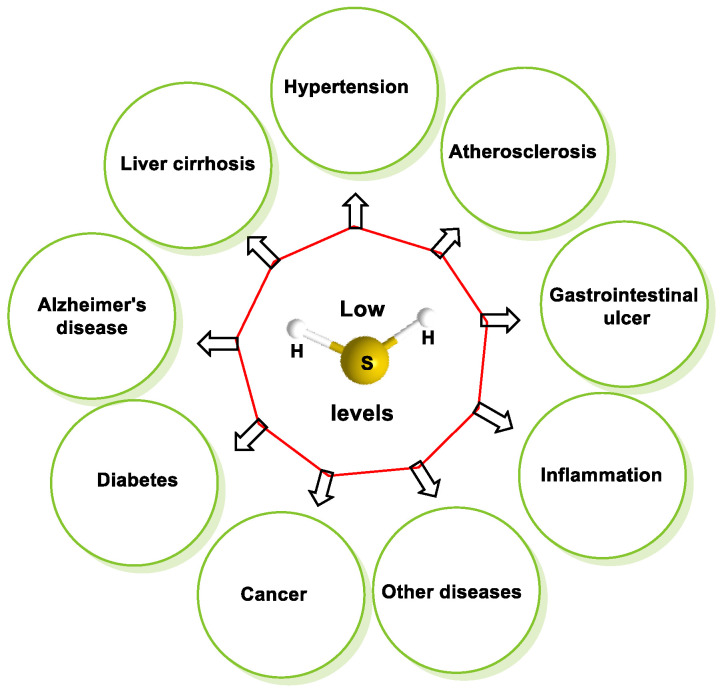
Examples of diseases related to low levels of H_2_S.

Endogenous H_2_S refers to the natural presence of this compound in the body because of regular metabolic processes in humans, animals and other organisms. Research conducted from the early 2000s to the present has revealed that endogenous H_2_S plays a critical role in regulating specific systems and processes in living organisms [82]. H_2_S has a high affinity for lipids, which makes it extremely lipophilic and facilitates its penetration of cell membranes, allowing it to enter various cell types. H_2_S plays a central role in the regulation of various physiological and pathological processes [83,84]. The potential consequences of decreasing H_2_S levels in the human body include the development and progression of various health conditions, including hypertension, atherosclerosis, gastrointestinal ulcers, cirrhosis of the liver, diabetes, inflammation, Alzheimer’s disease, cancer and other diseases [85]. The accompanying diagram provides a comprehensive representation of the biological mechanisms in human physiology that are controlled by endogenous H_2_S or show a response to pharmacological intervention with H_2_S or its derivatives (Figure 18).

**Figure 19:**
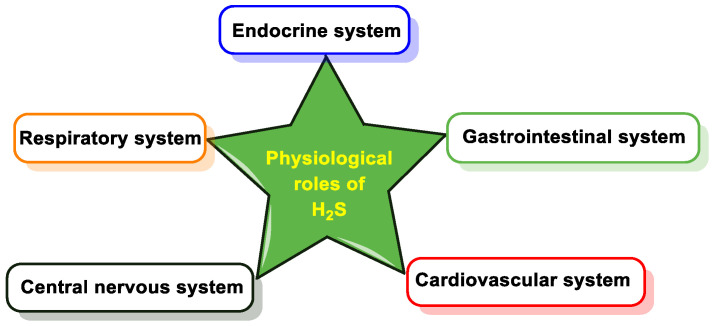
Physiological roles of H_2_S.

Among the variety of effects attributed to H_2_S, we can mention its participation in cardiovascular processes (in vasodilation, one of the first effects described), the central nervous system, the gastrointestinal system, the endocrine system, cytoprotection, etc. [86,87,88,89] (Figure 19).

### 6.1. Cardiovascular System

H_2_S plays a central role in modulating and regulating various signaling pathways involved in metabolism, cardiac function and cellular viability in mammalian organisms [90], and it has a significant effect on the cardiovascular system, blood vessels and blood components. Both mitochondrial activity and cellular metabolism are affected [21].

H_2_S has been found to be associated with hypertension, atherosclerosis and myocardial damage in the cardiovascular system. The potential efficacy of this compound in the treatment of hypertension may be due to its ability to induce vasodilation. Relaxation of the rat thoracic aorta, portal vein and mesenteric artery by H_2_S has been demonstrated, suggesting that hydrogen sulfide plays an important role in regulating contractility and blood pressure. In contrast, another study suggests that H_2_S exhibits vasoconstrictor properties at low concentrations, possibly through a mechanism that inhibits the activity of nitric oxide (-NO), a molecule also involved in contractility [91,92]. H_2_S may help patients recover from myocardial injury, particularly ischemia–reperfusion injury. Numerous cardiovascular diseases have been associated with H_2_S, suggesting a potentially broad applicability of H_2_S in the context of heart disease [90].

H_2_S exerts several effects on the cardiovascular system. These include attenuating ischemia–reperfusion injury to cardiac tissue, facilitating angiogenesis, relaxing smooth muscle cells and regulating blood pressure [93].

### 6.2. Gastrointestinal System

H_2_S is known to have a significant effect on reducing gastric mucosal damage and has the potential to act as a crucial mediator of gastrointestinal motility.

Insulin secretion and diabetes mellitus may be affected by H_2_S because the pancreas is among the targets of H_2_S [94,95,96]. In the pancreas, CSE is the main enzyme that converts cysteine to H_2_S. H_2_S concentration is elevated in response to the presence of pancreatitis, which is attributed to its proinflammatory effect. H_2_S administration contributes to chloride secretion, which aggravates certain types of gastritis [97]. H_2_S concentration increases when abdominal sepsis or endotoxemia occurs [98].

H_2_S has a protective anti-inflammatory effect on the gastrointestinal system in some types of gastritis and colitis [99,100,101]. H_2_S elicits both proinflammatory and anti-inflammatory responses in various models of inflammation [102]. The synthesis of H_2_S is markedly increased in colon ulcers, resulting in accelerated restoration of epithelial barrier integrity and healing of injured tissues [103].

### 6.3. Respiratory System

A study that investigated the relationship between H_2_S and pulmonary hypertension represents a pioneering achievement in the field of pathophysiology and H_2_S on a global scale [104]. In recent years, numerous studies have been conducted to investigate the involvement of H_2_S in the development of pulmonary hypertension. These studies have primarily focused on the administration of H_2_S to animal models suffering from chronic hypoxia.

H_2_S is implicated in the pathogenesis and therapeutic interventions of chronic obstructive pulmonary disease, as endogenous H_2_S is involved in the regulation of physiological functions of the respiratory system and pathophysiological alterations, such as chronic obstructive pulmonary disease, asthma, pulmonary fibrosis and hypoxia-induced pulmonary hypertension [105].

### 6.4. Nervous System

In addition, H_2_S exerts its action on important functions of the central nervous system and provides neuroprotective protection against oxidative stress. There is a belief that it has potentially protective properties against neurodegenerative diseases such as Parkinson’s disease, Alzheimer’s disease and Huntington’s disease [106]; spinocerebellar ataxia; and traumatic brain injury. It has been published that H_2_S levels are reduced in the brains of patients with Alzheimer’s disease compared to healthy individuals [107]. In the central nervous system, both sides of H_2_S are observed: it ameliorates ischemic lesions, but it leads to the aggravation of stroke.

### 6.5. Endocrine System

The variation in H_2_S concentration is related to a variety of endocrine disorders [108]. Understanding the effect of H_2_S concentration on the endocrine system is useful for the treatment of hypertension, diabetes and other diseases. H_2_S can affect the secretion of many hormones and participate in the onset and development of endocrine diseases. H_2_S can regulate hormone secretion through antioxidant stress and regulation of ion channels and protect endocrine organs. H_2_S can regulate glucose and fat metabolism through the pancreas, liver, adipose tissue and skeletal muscle [109].

### 6.6. Visual System

H_2_S protects retinal photoreceptor cells from light-induced degeneration. Deficiency of H_2_S or its substrates was found to be associated with ectopialentis, myopia, cataracts, optic atrophy and retinal detachment [110,111,112].

### 6.7. Age-Related Diseases

H_2_S is a reducing agent and can be metabolized by various oxidants in the human body. It counteracts oxidative species, such as reactive oxygen species (ROS and reactive nitrogen species (RNS), in the human body. The activation of antioxidant enzymes serves to limit the reactions of free radicals and thus protects against the harmful effects of aging [113].

H_2_S has several cytoprotective and physiological functions related to age-related diseases. Most notably, it serves as a potent antioxidant and gasotransmitter. Oxidative stress plays an important role in the development and progression of many age-related diseases [114].

### 6.8. H_2_S in Cancer

It is documented that several types of cancer such as colon, breast, ovarian and prostate cancers have higher levels of CBS, CSE or 3MST enzymes or synthesize greater amounts of H_2_S compared to adjacent non-tumor tissue [115,116,117,118].

The outcome of treating cancer cells with H_2_S donors depends on the concentration/dose, time, cell type and drug used. The effects of both natural and synthetic donors vary from potent cancer suppressors to promoters [40,119]. The administration of H_2_S donors to various cancer cell lines has been shown to produce cell death, with this effect being dependent on increasing concentrations of H_2_S. This indicates that H_2_S donors could represent a therapeutic potential as anticancer drugs. The combination of non-steroidal anti-inflammatory drugs with slow-release H_2_S donors has been shown to effectively inhibit the growth of human colon, mammary, pancreatic, prostate, lung and bone marrow cancer cells by promoting cell apoptosis via the activation of p38 MAPK [120].

### 6.9. H_2_S and Antimicrobial Resistance

The prevalence of antimicrobial resistance (AMR) is increasing and represents a major public health challenge [121]. Similar to mammalian organisms, bacterial cells also have three enzymes involved in the synthesis of H_2_S [122]. These enzymes are known as cystathionine-γ-lyase (CSE), cystathionine-β-synthetase (CBS) and 3-mercaptopyruvate sulfurtransferase (3MST) [123]. Bacteria have been shown to produce H_2_S as a cytoprotective agent in response to host-induced stress, such as oxidative stress and antibiotics [124]. Endogenously produced H_2_S stimulates ROS-scavenging enzymes and interferes with the Fenton reaction, reducing the amount of ROS produced by cells and promoting antibiotic tolerance [125]. Antibiotics such as quinolones, beta-lactams and aminoglycosides are more effective against bacterial pathogens in vitro and in mouse models when combined with small compounds that block a bacterial enzyme involved in the formation of hydrogen sulfide [126]. Therefore, it has been hypothesized that this messenger is a fundamental anti-antibiotic defense mechanism in bacteria [126,127]. In biofilms, persisting cells had significantly higher H_2_S content than active cells, supporting the notion that H_2_S is a critical component in bacterial biofilm formation [128]. However, not all significant pathogenic bacteria encode the H_2_S biosynthetic pathway. Pathogenic *Acinetobacter baumannii* bacteria do not produce endogenous H_2_S. By manipulating the sulfide content of *A. baumannii* with a H_2_S-releasing chemical, researchers showed that exogenous-H_2_S-sensitized *A. baumannii* was able to reverse acquired resistance to gentamicin [129]. It appears that the presence of exogenous H_2_S triggered a disruption of redox and energy balance that ultimately led to increased susceptibility to the lethal effects of antibiotics [126]. Therefore, it was hypothesized that H_2_S can be used as an antibiotic-potentiating and resistance-converting agent in bacteria that do not produce it themselves [129]. The recognition of H_2_S biogenesis is a promising focus for the development of antibacterial adjuvants to combat tolerance and resistance [130]. Influencing hydrogen sulfide-based defenses is a largely unexplored alternative to conventional antibiotic discovery.

## 7. Mechanisms of Action of H_2_S and Molecular Targets

**Figure 20:**
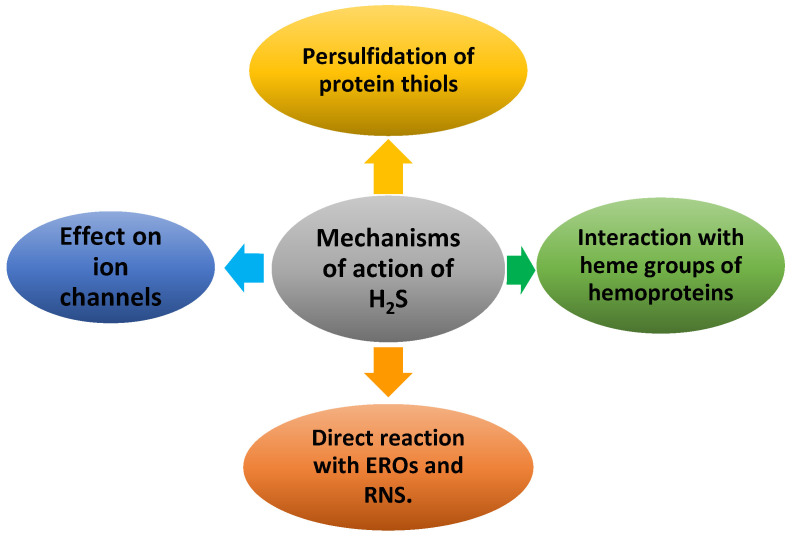
H_2_S signaling mechanisms.

The mechanisms through which H_2_S exerts its effects are not yet fully understood. However, there is sufficient consensus regarding four mechanisms of action or molecular targets (Figure 20).

H_2_S is of great importance as a secondary messenger that binds to specific target proteins to facilitate signal transduction, especially in the field of mammalian physiology. It is now known that the signal transduction function of H_2_S occurs through at least three main mechanisms: (i) interactions with metal centers [131], (ii) scavenging of ROS and RNS [132], (iii) S-persulfidation [6] and (iv) effects on ion channels.

### 7.1. Effect of H_2_S on Ion Channels

There is abundant literature on the effect of H_2_S on ion channels. For example, H_2_S opens ATP-dependent potassium channels [133,134] and modulates various types of calcium [135,136,137] and chloride channels [138].

Ion channels are proteins that form pores in the membranes of cells and organelles, having the function of regulating the flow of ions through them. H_2_S can act directly or indirectly on the channels. ATP-dependent potassium channels (K+_ATP_) are the most studied in terms of their interaction with H_2_S.

**Figure 21:**
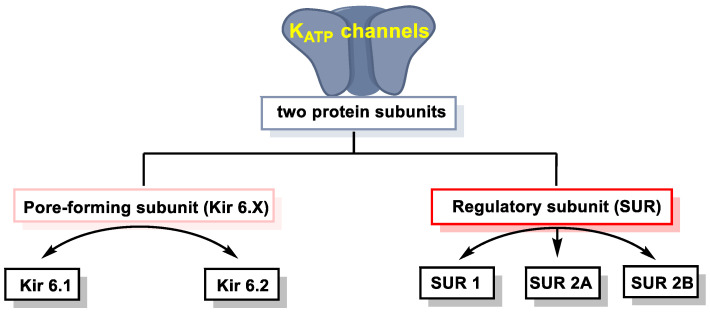
K_ATP_ channel subunits.

K+_ATP_ is a hetero-octamer consisting of four pore-forming subunits, Kir6.x, and four regulatory subunits, SURx, and their activity is inhibited by binding to ATP [139] (Figure 21).

Cysteine 43 of the Kir6.1 subunit is the target of H_2_S persulfuration. The interaction of the gas with the protein was accompanied by decreased ATP binding and increased binding of phosphatidyl inositol diphosphate, suggesting that the effect of the interaction with H_2_S decreases the affinity of the protein for ATP, thus activating the channel. Other authors showed with targeted mutagenesis experiments that the effect of H_2_S was only observed when the two subunits, Kir6.1 and rvSUR1, were present, but not with Kir6.1 alone [139]. They also identified cysteine residues in the regulatory subunit, cysteine 6 and cysteine 26, necessary for the effect.

H_2_S is unable to regulate the function of K+_ATP_ channels directly [140]. It has been observed that the cardioprotective effects of H_2_S partially disappear when K+_ATP_ channels are chemically blocked [141].

### 7.2. Direct Reaction of H_2_S with ROS and RNS

There is conflicting evidence for the influence of H_2_S on various organisms, mainly due to its toxic nature and its ability to scavenge reactive oxygen species (ROS) and thus mitigate oxidative stress [7,20]. Hydrogen peroxide, peroxynitrite, hypochlorite and the superoxide radical anion are examples of ROS and reactive nitrogen species (RNS) that can react with H_2_S. This means that it can inhibit the harmful effects of ROS and/or RNS on organic substances.

Recently, there has been increased interest in the study of H_2_S due to its toxic nature and its ability to protect bacteria from the harmful effects of oxidative stress induced by antibiotic therapy [142]. The process has the ability to render the redox centers of metalloenzymes inactive [143,144,145,146], causing DNA damage [147] and protein denaturation by disrupting disulfide bonds [148].

#### 7.2.1. Non-Radical Species (Two-Electron Oxidation)

**Figure 22:**
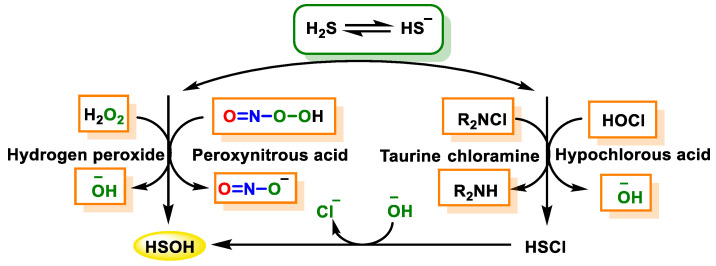
Oxidation of H_2_S by H_2_O_2_, ONOOH and HOCl. Formation of sulfenic acid (HSOH).

H_2_S reacts with two-electron oxidants such as hydrogen peroxide, peroxonitrous acid and hypochlorous acid and chloramines to transform into sulfenic acid (HSOH) which is an unstable intermediate (Figure 22).

The primary outcome of the chemical reaction between H_2_S and hydrogen peroxide (H_2_O_2_) is the formation of hydroxylthiol (HSOH). The final product consists largely of polysulfides, elemental sulfur and, in the case of excess oxidant, sulfate, and it depends on the initial ratios of hydrogen peroxide and H_2_S.

The direct reaction between peroxynitrous acid and HS^−^ involves a nucleophilic substitution of HS^−^, leading to the formation of HSOH and NO_2_^−^ as starting products. In the presence of an excess amount of H_2_S, HSOH further reacts with a second HS^−^, leading to the formation of HSS^−^/HSSH and other compounds.

It is likely that the interaction between hypochlorous acid and HS^−^ occurs through the formation of HSCl, which is subsequently subjected to rapid hydrolysis to produce HSOH. Chloramines, particularly RHNCl and R_2_NCl, exhibit lower reactivity but higher selectivity as oxidants compared to hypochlorous acid, as indicated in Table 1.

HSOH is the main product. Polysulfides, elemental sulfur and sulfate can all be produced by this reaction, but the exact composition of the final product depends on the ratio of hydrogen peroxide to hydrogen sulfide. It is noteworthy that the system exhibits the typical properties of a chemical oscillator. The oxidation of H_2_S can lead to the formation of a very reactive reductant: sulfoxylic acid. In many respects, H_2_S exhibits a reactivity profile similar to cysteine. It is a powerful nucleophile and, like thiols, can react with electrophiles as well as oxidants.

The oxidation process of H2S can lead to the formation of many compounds in which the sulfur atom can have oxidation states from −2 to +6. The oxidation products include several chemical compounds such as sulfate (SO_4_^2−^), sulfite (SO_3_^2−^), thiosulfate (S_2_O_3_^2−^), persulfides (RSS^−^), organic and inorganic polysulfides and elemental sulfur (Sn).

The oxidation pathways of thiols lead to the formation of sulfenic acids as transient intermediates. The main mechanism by which they are degraded is the formation of disulfide bonds in response to the presence of another thiol, and they are widely recognized for their instability [153,154].

#### 7.2.2. Radical Species (One-Electron Oxidation)

H_2_S can be oxidized to HS^−^/S^•−^ by a limited number of strong one-electron oxidizing agents, namely the hydroxyl radical (HO^−^), the carbonate radical (CO_3_^•−^), nitrogen dioxide (NO_2_^−^) and the peroxidase compounds oxoferryl I and II.

**Figure 23:**
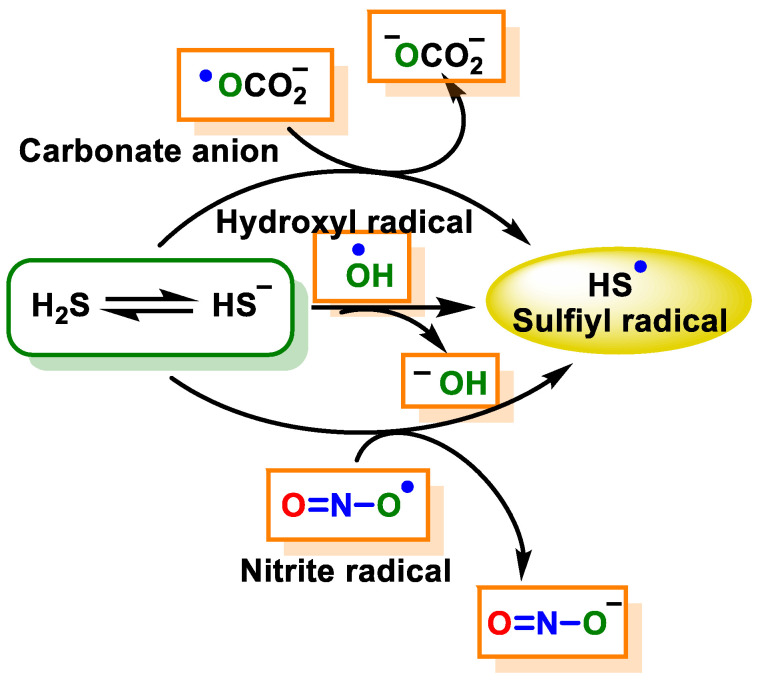
Oxidation of H_2_S and formation of the sulfiyl radical (HS^•^/S^•−^).

The initial product of the one-electron oxidation of H_2_S is the sulfyl radical (HS^−^/S^−^) (Figure 23).

**Figure 24:**
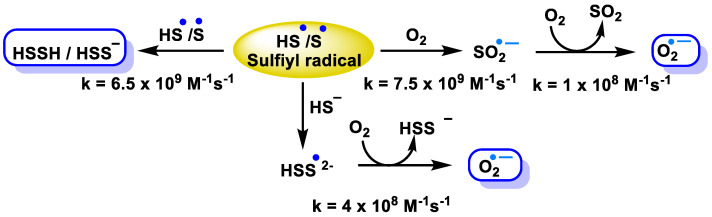
Chain reactions produced by the sulfiyl radical (HS^•^/S^•−^), which is an oxidant.

This particular radical exhibits oxidizing properties and is capable of undergoing reactions with a number of electron donors or hydrogen atoms. The compound is capable of undergoing a reaction with a secondary radical HS^−^/S^•−^, forming HSSH/HSS^−^ [155], or it can react with HS^−^ to form HSS^•2−^, which is a reducing radical that has the ability to undergo a reaction with oxygen that leads to the formation of the superoxide radical. HS^−^/S^•−^ can [155] also react with oxygen to form SO_2_^•−^, which again can react with oxygen to form a superoxide radical [156]. The one-electron oxidation of H_2_S has the ability to trigger oxygen-dependent free radical chain reactions that can lead to an amplification of the original oxidation species (Figure 24).

In addition to ROS and RNS, it is also necessary to consider reactive sulfur species, including hydropersulfides, polysulfides and H_2_S. The concept of RSS was postulated in 2001 [157], RSS that are produced under oxidative stress include RS^−^, sulfenic acids (RSOH), disulfides (RSSR), thiosulfinate (RS(O)SR), thiosulfonate (RS(O)_2_SR) and S-nitrosothiols (SNTs), the products of cysteine transformations, H_2_S and sulfane sulfur-containing compounds. SSRs that are produced under physiological conditions (without oxidative stress) are referred to in the scientific literature as “the first class of SSRs”. The “second class of SSRs”, on the other hand, “refers to species that are formed by the initial action of oxidative stress” [158].

H_2_S plays an important role in combating oxidative species such as ROS and RNS in the body. Filipovic, in 2012 [159], studied the reaction of H_2_S with peroxynitrite in vitro and in different cell models. The results showed that H_2_S can remove peroxynitrite with a second-order rate constant and that the reaction does not proceed through radicals. In this reaction, a new product is formed, which was characterized by spectral and computational studies as HSNO_2_ (thionitrate), mostly as sulfinyl nitrite HS(O)NO.

**Figure 25:**
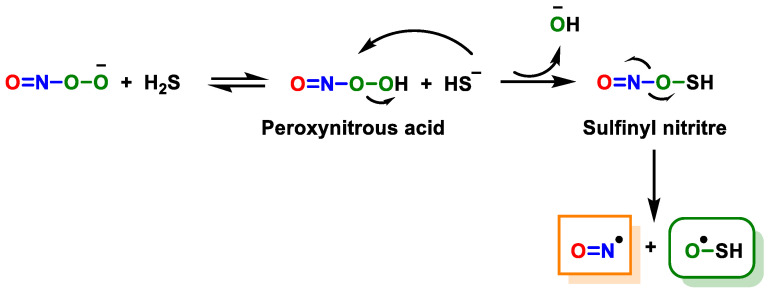
Reaction of H_2_S with peroxynitrite with formation of sulfinyl nitrite.

The ability of HS(O)NO to function as a nitric oxide (NO^•^) donor in response to pH and its ability to release NO^•^ in cellular environments have been successfully demonstrated. Therefore, H_2_S removal plays a role in modulating the chemical and biological effects of peroxynitrite. This process effectively suppresses the pro-apoptotic, oxidative and nitratative properties associated with peroxynitrite (Figure 25).

## 8. Persulfidation or S-Sulfhydration of Protein Thiols

**Figure 26:**
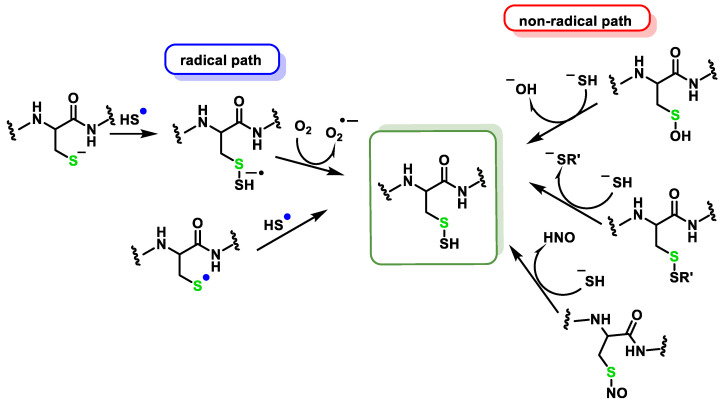
Mechanisms of protein persulfidation.

Sulfhydration of cysteine residues and nitration of tyrosine are H_2_S-induced post-translational modifications induced by H_2_S and RNS, respectively [160]. H_2_S is an important biological messenger molecule that transmits signals through the formation of persulfide bonds (SSH) in proteins or low-molecular-weight thiols [1,161,162]. S-Persulfidation is the process in which a thiol (R-SH) is converted into a perthiol (R-SSH). The modification of thiols to form persulfides is one of the mechanisms by which sulfide exerts signaling functions. Protein modification by persulfurization of cysteine residues is able to modulate the activity of different proteins. The formation of persulfides is associated with the body’s sulfur reserve [1]. The direct reaction of H_2_S with protein cysteines does not take place; for this reaction to occur, the presence of an oxidant is required. The proposed mechanism for the formation of persulfides is the reaction of sulfur with oxidized cysteines such as sulfenic acid (RSOH) or disulfide (RSSR) [163]. Persulfides can also be formed via radicals, through the reaction of the sulfhydryl radical (HS^•−^) with the RS^−^, although the low concentration of these species means that this reaction is of little biological relevance [148,164]. HS^•−^ can also react with a non-radical thiol to generate the radical anion RSSH^•−^, which gives up its unpaired electron to molecular oxygen to give the persulfide and superoxide radical anion [165] (Figure 26).

Persulfides are unstable and have an electrophilic character. They also retain the nucleophilic character of the original thiol, or even enhance it due to the presence of an adjacent sulfur containing unshared electron pairs, i.e., the α-effect [166,167,168]. It has been proposed that these compounds are responsible for the biological effects initially assigned to H_2_S [169].

One of the ways in which H_2_S functions as a messenger molecule is by sulfhydration of reactive cysteine residues of target proteins in a manner analogous to protein nitrosylation [1,28].

Due to a decrease in pKa and an increase in nucleophilicity of perthiols compared to thiols, S-persulfidation can affect the biological activity of proteins [170]. For example, the enzyme glyceraldehyde-3-phosphate dehydrogenase (GAPDH), which is primarily involved in glycolysis and gluconeogenesis, undergoes an activity shift after persulfidation to prevent cell death [170]. Persulfidation at K_ATP_ channels is a factor contributing to vasodilation caused by H_2_S [171].

**Figure 27:**
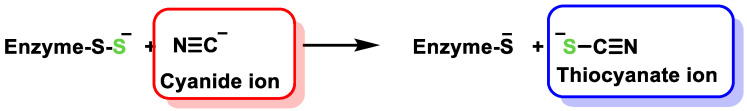
Hydropersulfide cyanolysis reaction.

The reaction of persulfides with cyanide gives thiols and thiocyanate (Figure 27).

**Figure 28:**
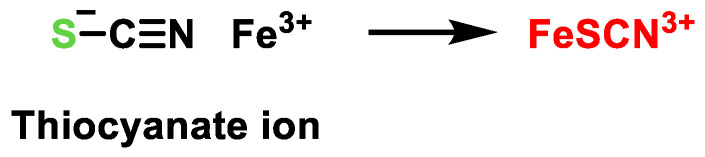
Persulfide detection reaction.

The cyanolysis reaction is a common reaction involving hydrosulfides and other sulfur compounds. This reaction serves as a reliable method to confirm the presence of the -SSH group on a protein. In addition, this reaction has characteristic properties of hydrosulfides and can be used for the detection of persulfides. The reaction between thiocyanate and ferric ions leads to the formation of a red complex that exhibits absorption at a wavelength of 460 nm. This complex can be accurately measured and quantified by spectrophotometric methods [172] (Figure 28).

## 9. Detection of H_2_S

The two deprotonated H_2_S species (HS^−^ and S^2−^) absorb in UV at 230 nm with molar extinction coefficients of 8 × 10^3^ and 4.6 × 10^3^ M^−1^ cm^−1^, respectively, at 25 °C, so the concentration of the predominant species (HS^−^) could be measured by absorbance [173]. In practice, their oxidation products generate interferences.

Several research groups have focused their efforts on the development of H_2_S detection probes. Classical instrumental methods for H_2_S detection include the following: (i) colorimetric and electrochemical assays, (ii) gas chromatography and (iii) sulfide precipitation [38,75,140,174,175]. These techniques often require complex sample processing. The results of these methods may differ due to the high reactivity of H_2_S [63,163,176]. The high sensitivity of fluorescence-based assays suggests that they could be potentially valuable in this area. However, the limited number of fluorescence techniques available today for the detection of H_2_S poses a challenge when it comes to monitoring this gas in biological samples in real time [75,177]. A major challenge is to develop molecular probes that can detect aqueous sulfides (H_2_S and HS^−^ at neuronal pH) in the presence of other thiols found inside most cells.

Numerous H_2_S detection techniques have been described, including spectrophotometric methods, where sulfide can be monitored by the formation of lead sulfide or methylene blue at 390 or 670 nm, respectively; fluorimetric methods, using fluorescein mercuric acetate; and polarographic methods with sulfide-specific electrodes, as well as by liquid or gas chromatography [178,179].

Another methodology used is the trapping of sulfide on ZnS particles by a reaction with zinc acetate. This technique is used in conjunction with other methods [14].

**Figure 29:**
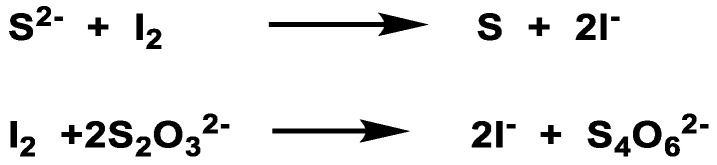
Classical iodometric titration.

Classical iodometric titrations are used to prepare a standard solution. H_2_S is first immobilized in zinc acetate to reduce its dispersion and then reacts with an excess of iodine in an acidic environment. The remaining iodine is subjected to titration with sodium thiosulfate, with starch added as an indicator (Figure 29).

**Figure 30:**
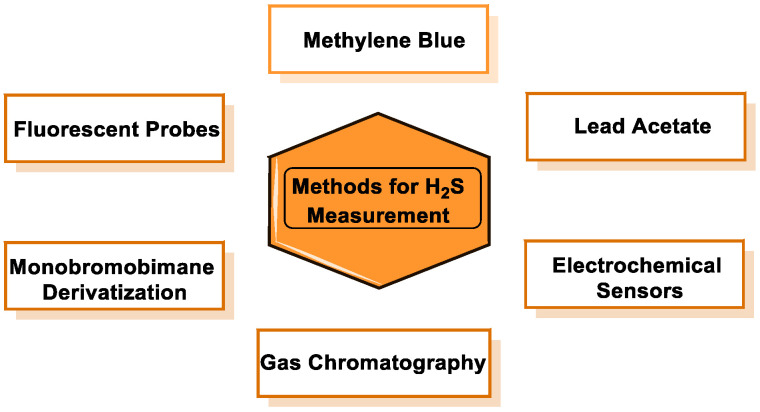
Methods for H_2_S measurement.

However, this method leads to errors due to the presence of other reductants. Numerous research groups have focused primarily on the development of probes for the detection of H_2_S. The approaches currently described for the detection of sulfur are usually based on its nucleophilicity or its reductive capacity, both of which are common to other thiols (glutathione and protein thiols) in biological studies and can easily mask the signal corresponding to sulfur. Figure 30 shows the most commonly used methods for H_2_S detection.

### 9.1. Lead Acetate

**Figure 31:**
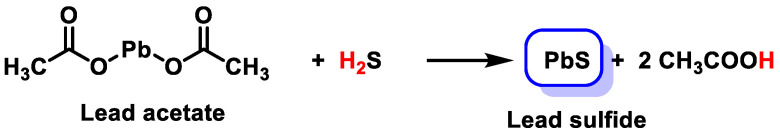
Hydrogen sulfide reacts with lead acetate to form a brown solid of lead sulfide (PbS).

The enzymatic synthesis of hydrogen sulfide can be traced back to a simple approach involving the use of lead acetate and the determination of the formation of lead sulfide (Figure 31), which is insoluble and can be detected by increasing turbidity at 390 nm.

When using this approach to calculate H_2_S concentrations, it is required to compare the results with a calibration curve created with already-known lead sulfide concentrations [180]. This approach provides semi-quantitative data and has relatively low sensitivity.

### 9.2. Methylene Blue Method

**Figure 32:**
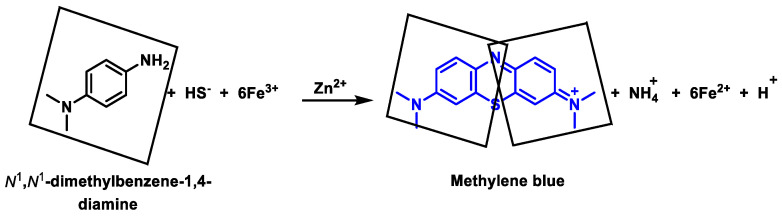
Reaction in the methylene blue method for sulfide detection.

Methylene blue is formed when an oxidizing agent, usually ferric iron, reacts with H_2_S_aq_ and N,N-dimethyl-p-phenylenediamine (N,N-dimethylbenzene-1,4-diamine) in an acidic environment (Figure 32).

The methylene blue formation reaction can be used to determine the H_2_S concentration. The concentration of methylene blue is determined at a wavelength of 670 nm and then compared with calibration curves generated with samples of known H_2_S concentrations subjected to comparable processing procedures [179,181]. This method also has a number of disadvantages.

### 9.3. Monobromobimane Derivatization

**Figure 33:**
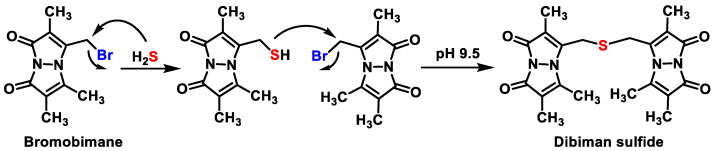
Bromobrimane reaction to obtain dibiman sulfide.

H_2_S undergoes a nucleophilic substitution reaction with monobromobimane, resulting in the formation of a bimane-substituted thiol compound. This biman-substituted thiol can further react with a second monobromobiman molecule, leading to the formation of dibiman sulfide. The fluorescence of dibiman sulfide can be observed upon its separation by high-performance liquid chromatography (HPLC) or mass spectrometry. This method has found wide use recently; however, the reaction rate is relatively slow (K ≈ 10 M^−1^ s^−1^ at pH 8) [182] (Figure 33).

### 9.4. Methods Based on the Reducing Capacity of H_2_S

**Figure 34:**
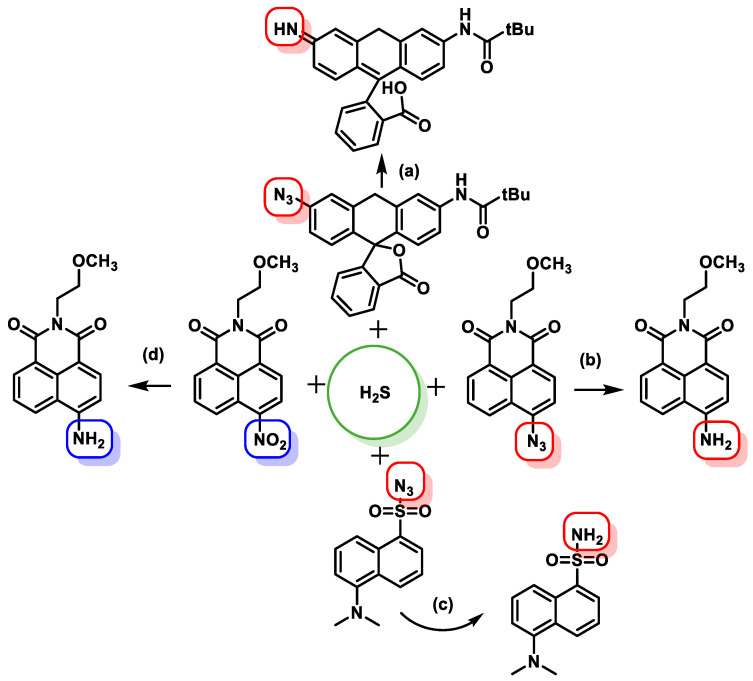
Fluorescent probes for the detection of H_2_S using the reduction of azide (**a**–**c**) or nitro (**d**) groups.

The sensitivity of fluorescent probes can be quite high. Certain probes, which have been described in detail, use nitro or azide derivatives of rhodamine, dansyl, coumarins or naphthylamides, which can be reduced by H_2_S to produce fluorescent amines (Figure 34).

In chemical synthesis, H_2_S is frequently used for the reduction of azido groups (N_3_) [183] and aromatic nitro groups [184] to aniline derivatives due to its strong reducing agent properties.

By attaching an N_3_ group to an SF1 rhodamine core, the research team led by Chang was able to produce a selective probe for the detection of H_2_S. Such probes exhibit strong selectivity for H_2_S over oxygen and nitrogen [185], two other reactive sulfur species that are physiologically important, and the fluorescent signal is released upon reduction to the amine. Using a similar strategy, Wang’s lab has developed a sulfonyl azidedansyl derivative, whose electrical and fluorescent properties are due to the different electronegativity of the azide and amine groups. H_2_S could be captured with a fluorescent probe to detect and visualize hydrogen sulfide. However, its slow kinetics are a disadvantage.

**Figure 35:**
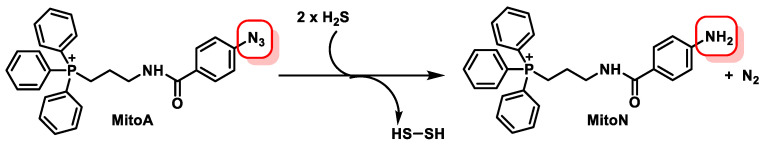
Reaction of MitoA with H_2_S to form MitoN.

To track changes in mitochondrial H_2_S content in living organisms, Mike Murphy’s team synthesized and characterized MitoA [186]. MitoA consists of an aryl azide coupled with a lipophilic triphenylphosphonium cation (TPP). In living organisms, the TPP cation causes MitoA to accumulate in the mitochondrial structures of the cell. The arylazide group forms MitoN, an arylamine species, when it interacts with H_2_S (Figure 35).

Therefore, the extent to which MitoA is converted to MitoN serves as an indicator of the amount of H_2_S in the mitochondria of a living organism. Detection and quantification of these chemicals in tissues can be performed with high sensitivity by liquid chromatography–tandem mass spectrometry (LC-MS/MS), using deuterated internal standards for accurate measurement.

Certain electrochemical techniques utilize the inherent reducing capability of sulfide as a fundamental principle. A polarographic technique based on the oxidation reaction between sulfide and ferricyanide can be used [175].

### 9.5. Methods Based on Nucleophilicity

H_2_S is considered a nucleophile that usually occurs as HS^−^ under physiological pH conditions. Consequently, it exhibits stronger nucleophilic activity than various other thiols in the cellular environment, which are predominantly present in their protonated form (RSH). The observed difference indicates specific advantages in terms of the nucleophilicity of sulfur compared to thiols. For the selective detection of H_2_S, it is crucial to distinguish H_2_S from other nucleophilic compounds present in biological systems, especially thiols such as cysteine and glutathione. In a theoretical context, it is plausible to classify H_2_S as an unmodified thiol that has the potential to perform two nucleophilic attacks. In contrast, other thiols, such as cysteine, are capable of performing only a single nucleophilic attack. In view of these considerations, probes containing electrophilic functions capable of transforming in the presence of H_2_S have been described.

HS^−^ is known for its strong nucleophilic properties, which allow it to readily bind to electrophilic sites in luminescent compounds such as cyanine dyes. This approach has been used in the development of radiometric H_2_S sensors, where fluorescence emission is altered by disrupting an extended pi system [187,188,189].

**Figure 36:**
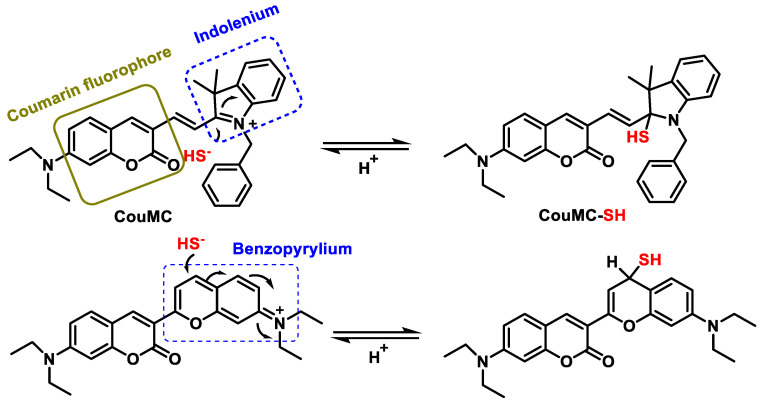
Structures of ratiometric H_2_S probes that function by disrupting the conjugated p-system within a fluorophore. Also shown is the process by which these probes react with H_2_S.

This review introduces a novel radiometric fluorescence probe called CouMC, which is described in a recently published research article. The functionality of this probe is based on the process of selective nucleophilic addition of HS^−^ to a merocyanine derivative in a near-neutral pH environment. In addition to its ability to rapidly and specifically detect H_2_S, this probe also shows potential for the selective visualization of H_2_S in the mitochondria of living cells (Figure 36).

To construct the CouMC probe, an ethylene group was used to link a coumarin fluorophore to an indole block. By aligning with the electrically positive benzopyrilium moiety of the fluorescent probe, H_2_S can be distinguished from biothiols and other biological products. This allows the probe to detect H_2_S based on a flavilium derivative.

**Figure 37:**
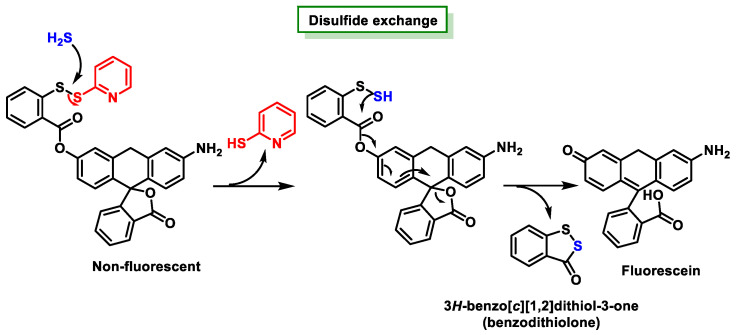
Fluorescent probe for the detection of hydrogen sulfide on the basis of H_2_S-mediated benzodithiolone formation.

The sensitivity of fluorescent probes can be of a considerable order of magnitude. The Xian research group postulated that the introduction of a probe with two electrophilic cores could potentially lead to selectivity for H_2_S, prompting them to investigate this particular property. The synthesis of a fluorescein ester of thiosalicylic acid was achieved by introducing a thiopyridyl disulfide functionality into the thiol group. This result was achieved by a synthesis process. Active disulfides can undergo disulfide exchange reactions with thiols and H_2_S. It is important to note that further rearrangement can only take place with the disulfide intermediate generated from H_2_S. The rearrangement of the ester occurs because of an intramolecular nucleophilic attack on the carboxyl carbon atom, resulting in the release of benzodithiolone and a fluorophore [75] (Figure 37).

**Figure 38:**
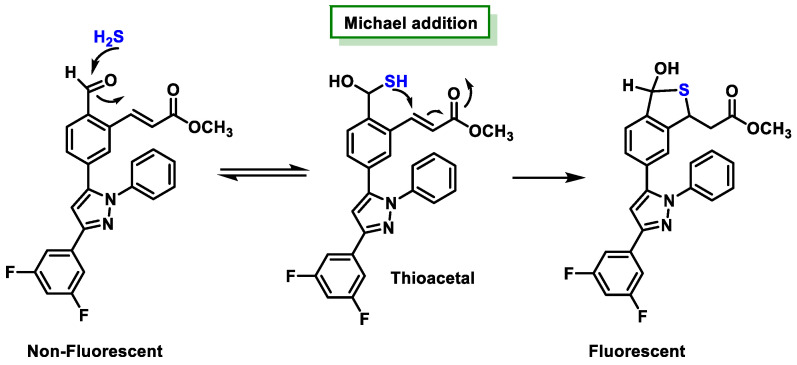
Fluorescent probes and reaction of methyl (E)-3-(5-(3-(3,5-difluorophenyl)-1-phenyl-1H-pyrazol-5-yl)-2-formylphenyl)acrylate with H_2_S.

Aldehydes and acrylates are two examples of electrophilic centers that have been used in the development of probes. Hemithioacetal is formed by the addition of hydrogen ions to the aldehyde compound. Subsequently, in a neutral pH aqueous solution, the nucleophilic behavior of S is observed; S undergoes an intramolecular Michael addition, specifically adding to the beta position of the α,β-unsaturated ester. The process described above leads to a cyclic thioacetal compound. The results of this research represent a significant advance for Chuan’s research team, as they have effectively visualized the enzymatic production of H_2_S in living cell systems [190] (Figure 38).

### 9.6. Methods Based on the Ability to Bind Metal Cations

**Figure 39:**
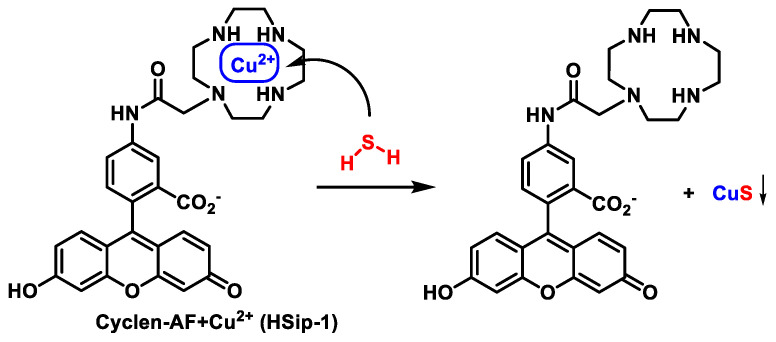
Use of azamacrocyclic copper(II) ion complex chemistry to modulate fluorescence in the fluorescent probe for the detection of H_2_S, known as HSip-1.

Another property that can be exploited for the detection of H_2_S is its remarkable affinity for metals. Novel probes consisting of a fluorophore coupled with Cu^2+^ have been developed. The precipitation of CuS and subsequent increase in fluorescence are the result of H_2_S binding to the copper ion [191] (Figure 39).

## 10. H_2_S Donors

There is an urgent need for the development of novel chemical tools that facilitate the controlled release of H_2_S to study its biological activities for research purposes and potential therapeutic applications. This need arises from the frequently observed low concentrations of endogenous H_2_S, which pose a challenge in studying its biological effects. Different categories of hydrogen sulfide donors can release hydrogen sulfide at different rates. Since a synthetic source of H_2_S is needed, chemists have developed chemical tools to analyze H_2_S in biological contexts.

Given the problems associated with the direct administration of H_2_S gas in various biological contexts, inorganic sulfide salts such as sodium (NaSH) and sodium sulfide (Na_2_S) are often used as alternative sulfide sources. Potential problems associated with these salts include their susceptibility to oxidation, their volatility and/or shortened effective residence time, and the rapid release of hydrogen sulfide upon dissolution, which can be challenging in some contexts [192]. To address this problem, researchers have developed and studied small H_2_S donors that release H_2_S at a controlled rate, like enzymatic H_2_S synthesis [193]. Currently, there is a wide range of chemicals that are commonly used as donors of H_2_S. These compounds include aryl isothiocyanates [194], phosphinodithioates [195], thioacids [196]/amides [197], dithiolethiones [198], thiolysis of protected trisulfides [199] and persulfides [200]. In addition, the emission of carbonyl sulfide (COS) from thiocarbamates, followed by its conversion to H_2_S by the action of carbonate anhydrase [201], is also considered to be a component of these donors.

### 10.1. Sulfur Salts

**Figure 40:**
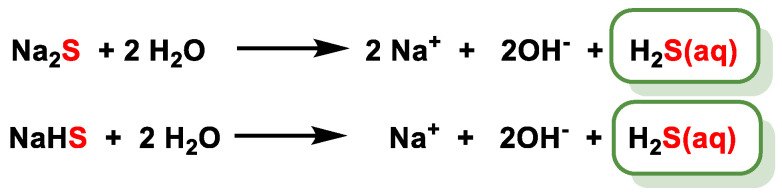
Sodium acid sulfide and sodium sulfide spontaneously release H_2_S.

Inorganic salts, such as sodium acid sulfide (NaHS) and sodium sulfide (Na_2_S), were historically the first compounds used and are still the most widely used to generate H_2_S. These salts are very soluble in water and upon dissolution release a large amount of H_2_S quickly, and for this reason, they are called fast-releasing donors (Figure 40).

More recently, slow-releasing donors that gradually release small amounts of H_2_S have been developed. These are usually organic compounds, and their effect more closely resembles a physiological situation, where the gas is released at a low but constant rate [202]. One of the most widely used slow releasers is GYYY4137 [195]. In addition, peptide-based releasers capable of releasing H_2_S in a more controlled manner are being explored [203].

### 10.2. Natural Donors

The human body is able to convert sulfur compounds contained in some foods such as garlic, onions, mushrooms and selected edible legumes and fruits into hydrogen sulfide through chemical or enzymatic processes. The compounds isolated from natural products are polysulfides substituted with allyl residues. The garlic derivatives diallyl sulfide, diallyl disulfide and diallyl trisulfide (DAS, DADS and DATS) are capable of releasing H_2_S in the presence of GSH.

There has long been speculation about the possible health benefits associated with the consumption of garlic.

**Figure 41:**
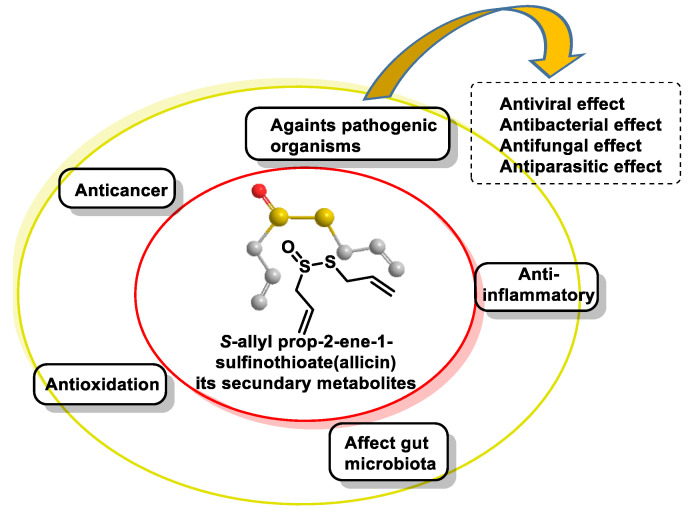
The biological functions of allicin and its secondary metabolites.

The major bioactive compound in freshly pressed garlic and certain other Allium plants is allicin [204]. Allicin is a naturally occurring compound that is chemically synthesized during the mechanical crushing of a garlic clove [205]. The compound exhibits inherent instability and is converted into many secondary metabolites that possess bioactive properties. Allicin and its secondary metabolites are a class of organosulfur compounds (OSCs) that exhibit various physiological functions, such as anticancer properties, activity against pathogenic organisms, modulation of the gut microbiota and antioxidant and anti-inflammatory effects. Their effects on pathogenic organisms include antibacterial, antifungal, antiviral and antiparasitic activities [206] (Figure 41). Numerous studies have demonstrated the following beneficial effects of garlic on cardiovascular health: (i) reducing blood pressure, (ii) reducing blood cholesterol levels and aggregation of platelets and (iii) reducing oxidative stress. S-allyl-l-cysteine (SAC) is proposed to be responsible for the cardioprotective effects of garlic.

**Figure 42:**
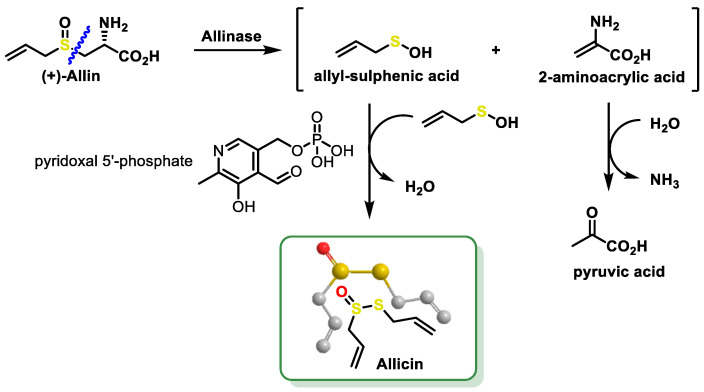
Enzymatic conversion of allin to allicin. Two molecules of 2-propenesulfenic acid interact to form allicin and remove water.

When garlic cloves are crushed or chopped, the enzyme allinase, which is stored in the vacuoles, is released. When it comes into contact with the cytosolic allicin, it converts it into a series of thiosulfinates, of which allicin is the best known (Figure 42).

**Figure 43:**
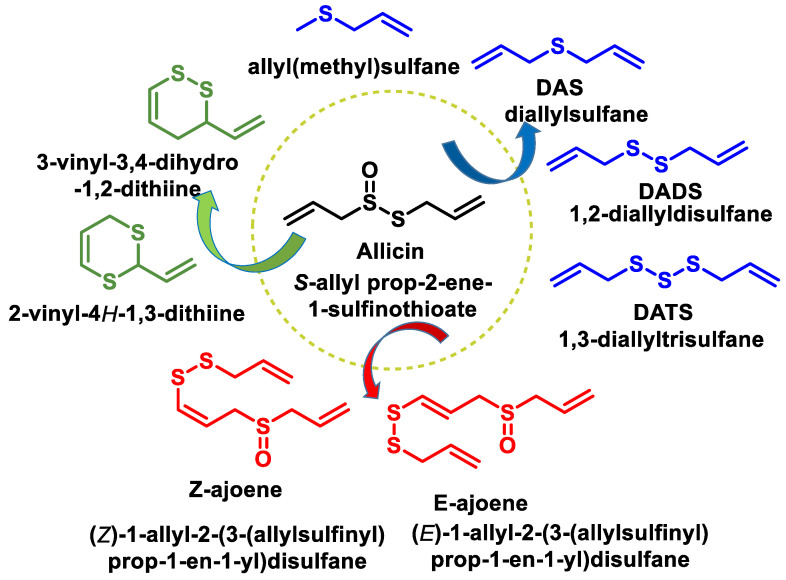
Organosulfur compounds derived from allicin.

The chemical compound allicin exhibits a high degree of instability, as it is susceptible to decomposition and subsequent rearrangement, which leads to the formation of various organosulfur compounds (Figure 43).

Ajoene is a colorless liquid containing a sulfoxide group and a disulfide group and is found as a mixture of up to four stereoisomers and two geometric isomers (E-Z) due to the chirality of sulfoxide (R-S). This chemical that gives garlic its distinctive aroma and flavor is released when the cloves are mechanically crushed or cut. To synthesize ajoene, allicin is dissolved in various solvents, such as edible oils. It has antioxidant properties and thus prevents the formation of superoxides. In addition, it shows antithrombotic and antiviral effects, especially against vaccinia, herpes simplex, rhinovirus, human parainfluenza virus and vesicular stomatitis virus. In addition, ajoene has been observed to have an inhibitory effect on integrin-mediated viral mechanisms in the context of HIV infections. In addition, this molecule has shown antibacterial and antifungal properties, particularly against *Candida albicans* and *Tinea pedis* infections.

**Figure 44:**
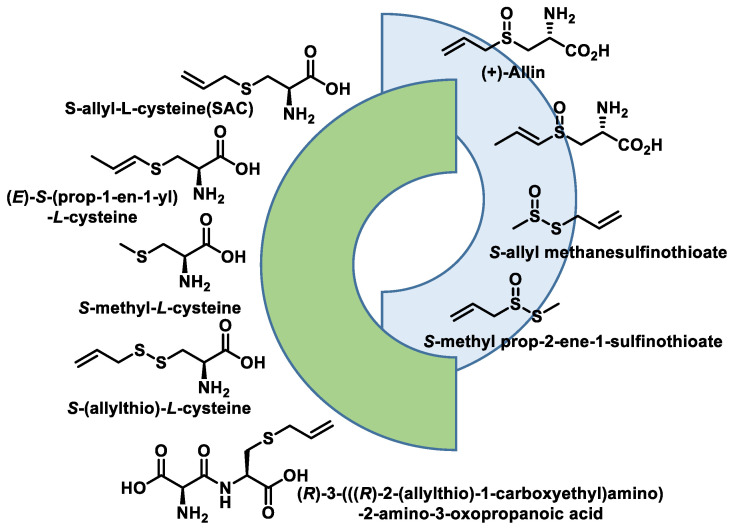
The main compounds found in intact garlic cloves.

The chemical composition of the garlic juice produced by the mechanical crushing of the garlic cloves differs significantly from that of the intact garlic clove, particularly with regard to the content of organosulfur components. Unprocessed garlic consists mainly of sulfur derivatives (Figure 44).

**Figure 45:**
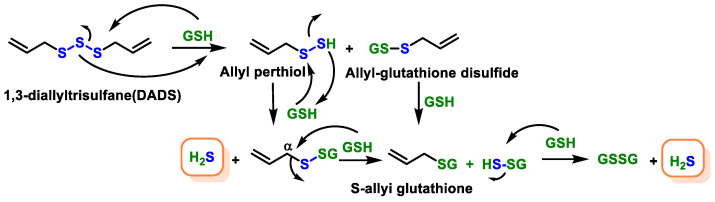
Proposed mechanism of H_2_S release from trisulfide in the presence of GSH by initial GSH attack on sulfur and a second GSH attack on α-carbon with the formation of S-allyi glutathione.

The proposed mechanism of H_2_S release from DADS by GSH attack of sulfur to form an allyl perthiol is shown in Figure 45. Similarly, red blood cells rapidly released H_2_S from diallyl disulfide (DADS) under oxygen deprivation and in the presence of glutathione [199].

The identification of DATS signifies the early recognition of a garlic-derived chemical that possesses vasoactive properties as well as a marked ability to selectively inhibit the proliferation of cancer cells [207]. Garlic-derived chemical compounds are commonly known as precursors of H_2_S during the absorption and metabolization process in the circulatory system.

The antiviral potential of garlic and its CSOs has been demonstrated in both in vivo and in vitro studies against a variety of viruses, including those belonging to the families *Adenoviridae*, *Arteriviridae*, *Coronaviridae*, *Flaviviridae*, *Flaviviridae*, *Herpesviridae*, *Orthomyxoviridae*, *Picornaviridae*, *Paramyxoviridae*, *Poxviridae*, *Rhabdoviridae* and *Retroviridae*. Blocking viral entry and fusion into host cells; inhibiting viral RNA polymerase, reverse transcriptase and viral replication; and enhancing the host immune response are the primary mechanisms by which garlic and its CSOs exert their antiviral activity. Garlic and its CSOs were also responsible for enhancing the host immune response. Further research is needed to better understand the properties of garlic and its active compounds, known as CSOs, in relation to their role in antiviral therapy. This means that additional research needs to be conducted focusing on the pharmacokinetics and clinical aspects of the therapeutic effect of garlic.

**Figure 46:**
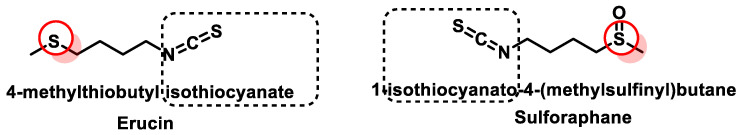
Natural sulfur compounds present in plants that have been associated with the formation of hydrogen sulfide.

Other natural sulfur compounds extracted from other plants are isothiocyanates (or sulforaphane), present in cabbages such as broccoli and cauliflower, and erucin, present in seeds and leaves of rocket [101,208], (Figure 46).

### 10.3. Synthetic H_2_S Donors

**Figure 47:**
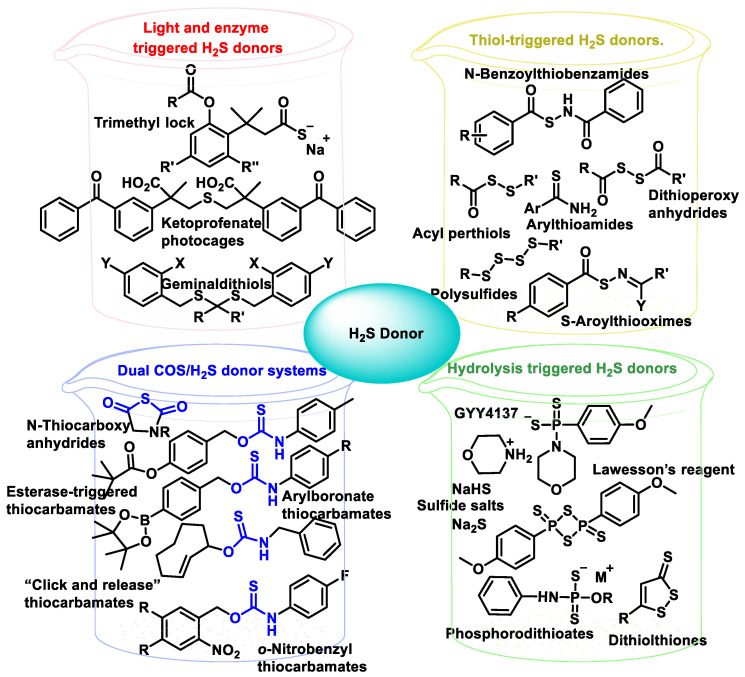
Organic H_2_S donors and the mechanisms by which they produce H_2_S.

Organic hydrogen sulfide donors, unlike inorganic sulfide salts, are capable of producing a continuous and regulated release of hydrogen sulfide in concentrations comparable to endogenous conditions. Many different types of organic H_2_S donors have been developed, and the methods by which they produce H_2_S are as diverse as possible. To advance the development of novel H_2_S-releasing donors, scientists have begun to alter the chemical compositions of extensively characterized substances known for their sulfur-releasing properties. Examples include donors activated in response to hydrolysis; to endogenous species such as thiols, ROS and enzymes; and to external stimuli such as photoactivation and bioorthogonal chemistry. Another possibility for the release of H_2_S is the use of the catalyzed hydrolysis of carbonyl sulfide (COS) by carbonic anhydrase. Figure 47 shows the published H_2_S donors, grouped according to their activation mechanism [209,210,211].

### 10.4. H_2_S and ^•^NO Hybrid Synthetic Donor

**Figure 48:**
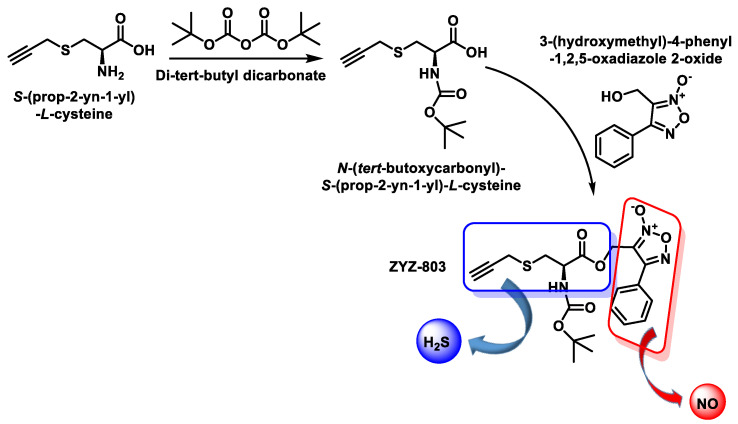
Chemical synthesis of ZYZ-803.

It is synthesized from S-(prop-2-yn-1-yl)-L-cysteine by reaction with di-tert-butyl dicarbonate and subsequent reaction of N-(tert-butoxycarbonyl)-S-(prop-2-yn-1-yl)-L-cysteine with 3-(hydroxymethyl)-4-phenyl-1,2,5-oxadiazole 2-oxide (Figure 48).

ZYZ-803 is a synthetic H_2_S and ^•^NO donor, in vitro and in vivo, and its potency appears to be higher than that of the H_2_S and/or ^•^NO donor. ZYZ-803 also stimulates H_2_S production from CSE and ^•^NO production from eNOS [212,213].

Despite the remarkable development and study of H_2_S donors, there is an absence of compounds that can address all the requirements for the perfect H_2_S donor in clinical studies.

### 10.5. Mitochondrial H_2_S Donors

**Figure 49:**
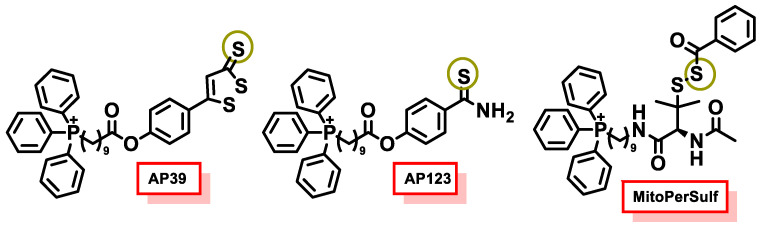
Mitochondrial H_2_S donors.

AP39 [214], AP123 [215] and MitoPerSulf [216] as new donors of slowly released hydrogen sulfide to the mitochondria are shown in Figure 49.

H_2_S donors with a slow mitochondrial release rate, such as AP39 and AP123, are >1000 times more potent than Na_2_S against hyperglycemia-induced oxidant production and also have a beneficial effect on cellular bioenergetics in endothelial cells [215].

**Figure 50:**
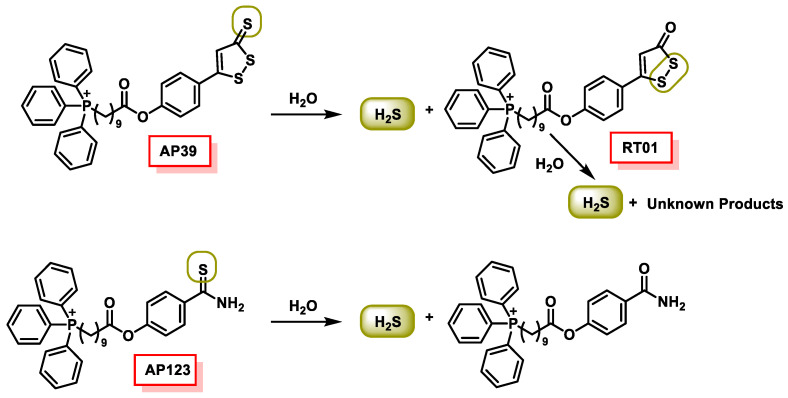
H_2_S release mechanism by AP39 and H_2_S release mechanism by AP123.

The thiocarbonyl group of the 1,2-dithiole-3-thione of AP39 is hydrolyzed to form the corresponding 1,2-dithiole-3-one (RT01) and releases H_2_S. RT01 undergoes further hydrolysis to release H_2_S and generate a series of unknown products. The mechanism of H_2_S release by AP123 involves the hydrolysis of the thiocarbonyl group of the thiobenzamide to form the corresponding amide and releases H_2_S (Figure 50).

**Figure 51:**
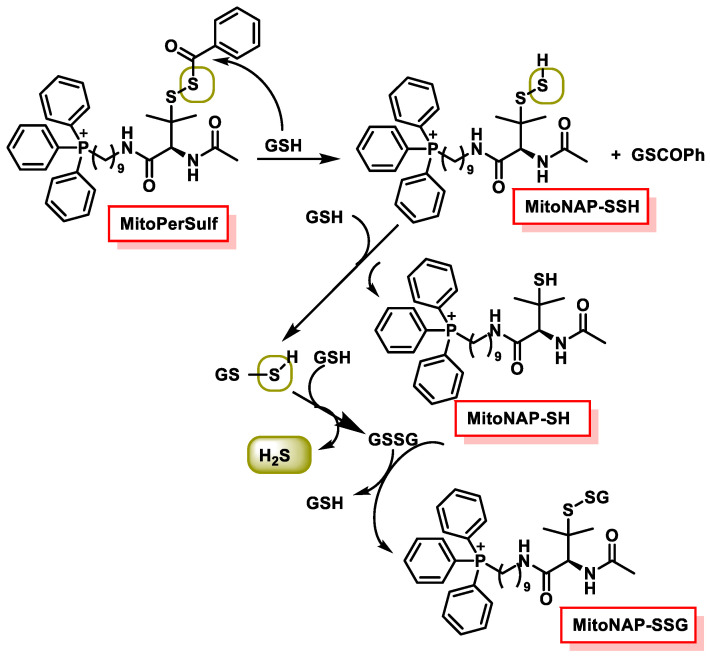
Reaction mechanism of thiol-dependent H_2_S release by MitoPerSulf. Reactions with GSH are shown.

MitoPerSulf is rapidly taken up by the mitochondria, where it reacts with endogenous thiols to generate a persulfur intermediate that releases H_2_S. MitoPerSulf reacts with thiols such as GSH to rapidly form persulfide, MitoNAP-SSH and GSCOPh. In the presence of excess GSH, GSSH forms H_2_S by forming GSSG which can react with MitoNAP-SH to form MitoNAP-SSG (Figure 51).

The facilitation of the internalization of MitoPerSulf into the mitochondria is enhanced by the incorporation of the triphenylphosphonium group (TPP), which has a special affinity for the mitochondria. Upon entry into the intended environment, the benzoyl thioester is cleaved due to its interaction with the thiols, resulting in the formation of the labile persulfide compound known as MitoNAP-SSH. The mitochondrial thiols play a crucial role in the persulfidation processes associated with each persulfide. The previously described persulfides show a remarkable propensity to generate hydrogen sulfide and to form disulfides when they interact with other thiols.

## 11. Hydrogen Sulfide as a Therapeutic Agent

### 11.1. Garlic Source of H_2_S Supplements

Garlic has a unique aroma and is a popular ingredient in many dishes. It contains several bioactive components such as polysaccharides, organic sulfides, saponins and phenolic chemicals. Garlic contains organic sulfides such as allicin, alliin, diallyl sulfide, diallyl disulfide, diallyl trisulfide, ajoene and S-allyl cysteine, which are its main bioactive constituents. The bioactive components of garlic have numerous biological effects, such as reducing inflammation, protecting the cardiovascular system, fighting cancer, preventing diabetes, reducing obesity, protecting the nervous system, preventing kidney damage and fighting bacteria and fungi. Through a mechanism involving the reduction of thiols in or on the cell membrane, human red blood cells or rat aortic rings can convert garlic-derived organic polysulfides into hydrogen sulfide [199].

The formation of H_2_S during the decomposition of organic polysulfides is enhanced by the presence of allylic substituents and an increasing number of sulfur atoms attached to the molecule. In view of the new information obtained, it is conceivable that changes can be made to the compound S-allylcysteine (SAC) found in garlic. The result was a novel molecule called S-propargyl cysteine (SPRC), which is capable of releasing H_2_S. It was successfully synthesized and proved to be resistant to oxidation in the presence of air [217]. The administration of SPRC (ip) showed a reduction in cognitive impairment caused by intracerebroventricular injection of LPS in rats. The administration of SPRC effectively reduced the lipopolysaccharide (LPS)-induced decrease in H_2_S levels in the hippocampus of rats.

The proven anticancer effect of allicin and its secondary metabolites suggests promising prospects, even if the practical application of this compound as a pharmaceutical drug is still a long way off. Further experiments are needed to improve the effectiveness of this treatment in fighting cancers of the digestive tract. Garlic is characterized by its non-toxicity or relatively low toxicity. Further studies are needed to explore the potential effects of fermentation or heat treatment of garlic, as these processes may affect the physiological functions and safety aspects of garlic. Additional clinical studies are needed to validate the putative therapeutic benefits of garlic in human cohorts, with particular attention to thoroughly assessing any adverse effects and ensuring overall safety.

### 11.2. Sulfur Drugs and Their Therapeutic Potential

Functional groups produced from sulfur exhibit a wide range of pharmacological properties and serve as valuable resources for the development of new therapeutic drugs. Pharmaceutical compounds containing sulfur in their chemical composition have the potential to either (i) release H_2_S or (ii) affect its endogenous synthesis. The ability of pharmaceutical compounds to release H_2_S has the potential to increase their biological efficacy and provide additional therapeutic benefits, but it can also lead to unfavorable outcomes. The action of sulfur groups and the emission of H_2_S from the active ingredient may affect the pharmacological activity of routinely used drugs.

**Figure 52:**
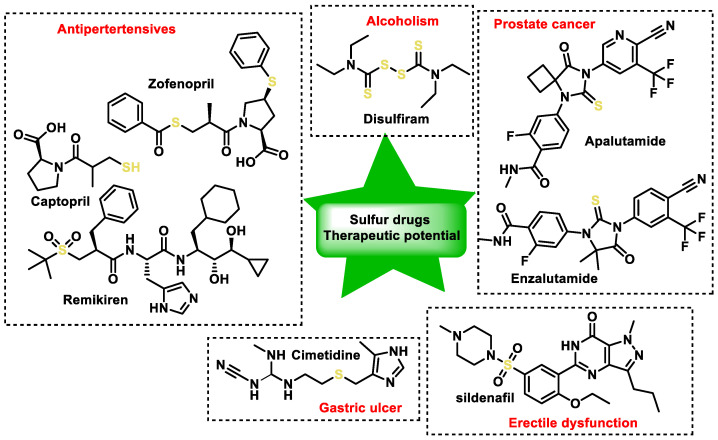
Examples of approved and used drugs containing sulfur-containing functional groups or residues and therapeutic applications.

Drugs with various therapeutic purposes, such as antihypertensive drugs, antibacterial agents, analgesics, anticancer drugs and anti-inflammatory compounds, may contain detectable amounts of sulfur (Figure 52).

**Figure 53:**
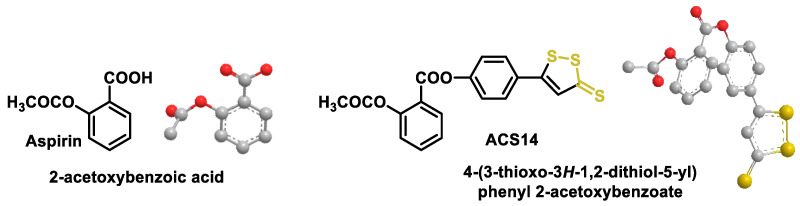
Structural formula of 2-acetoxybenzoic acid and 4-(3-thioxo-3H-1,2-dithiol-5-yl) phenyl-2-acetoxybenzoate.

It is assumed that the activities of these compounds are related to their ability to release hydrogen sulfide [218]. Drugs that are capable of releasing H_2_S have been synthesized as a strategy to enhance their effects or to reduce the adverse effects of treatments. For example, a H_2_S-releasing derivative of aspirin was effective in protecting the stomach mucosa against gastric damage from regular aspirin in rats [219]. Figure 53 shows the H_2_S-releasing aspirin derivative (ACS14) and aspirin.

It has been postulated that H_2_S in combination with non-steroidal anti-inflammatory drugs (NSAIDs) may act as a mediator inducing an anti-inflammatory response. Studies have shown that non-steroidal anti-inflammatory drugs (NSAIDs) that release H_2_S have increased efficacy and/or improved safety characteristics. Recent evidence has shown that gaseous mediators can improve blood circulation; attenuate oxidative stress; protect gastrointestinal mucosa from damage; enhance the anti-inflammatory properties of non-steroidal anti-inflammatory drugs (NSAIDs), and promote the resolution of inflammation, angiogenesis and epithelialization [220].

**Figure 54:**
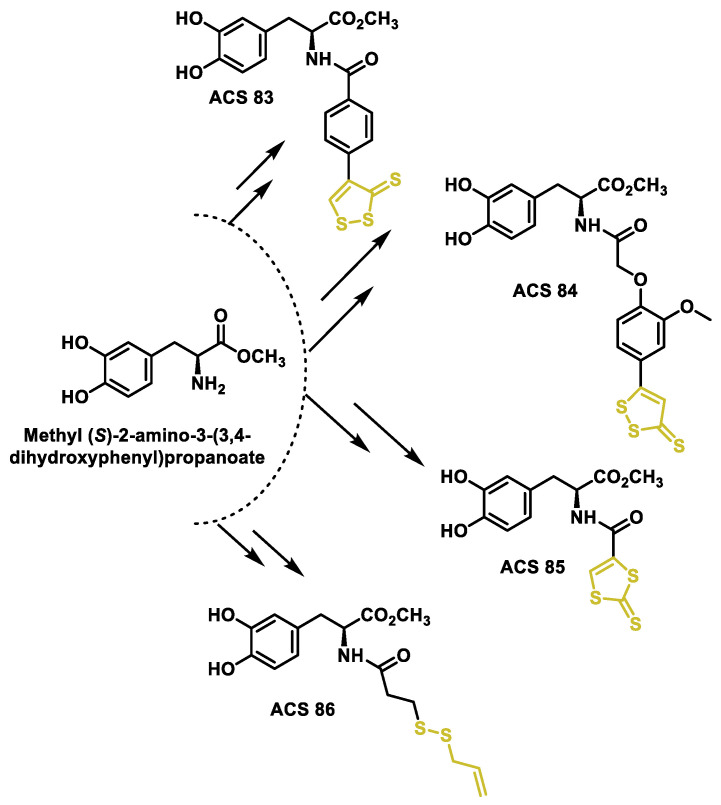
The structure of the H_2_S-releasing agents derived from l-DOPA: ACS83, ACS84, ACS85 and ACS86.

A diclofenac derivative showed a greater anti-inflammatory effect than diclofenac [220]. H_2_S-releasing l-DOPA derivatives (ACS83, ACS84, ACS85 and ACS86) (Figure 54) were synthesized and studied for the treatment of Parkinson’s disease [221].

### 11.3. Balneotherapy and H_2_S

Balneotherapy in sulfurous waters, although effective on its own, is even more effective when applied in conjunction with a pelotherapy treatment with peloids with sulfurous components or with hydro-hypnotherapy with sulfurous water. Pelotherapy, in chronic rheumatic pathology, consists of local application by means of plasters on the affected areas, or the immersion of the whole body in the peloid at a high temperature (39–48 °C), for 20–30 min, during 12–15 days of treatment. In natural antioxidant peloids (NAPs), a special clay is combined with sulfurous mineral medicinal water (AMmS), rich in H_2_S (13 mg/L), which is used in rheumatological, vascular and dermatological treatments, with excellent medical results, especially in the elderly. Sludges made with AMmS, with a recognized antioxidant capacity, due to the effective presence of H_2_S, are those that have been shown to have the greatest therapeutic capacity.

Studies carried out solely with sulfurous waters in balneotherapy, pelotherapy and drinking water in patients with osteoarthritis/arthrosis (OA) reveal that these waters increase the levels of H_2_S in plasma after the three types of treatment together [222] and that in general they have an antioxidant effect [223,224] and improve patient mobility and overall quality of life.

## 12. Conclusions

The identification of the endogenous synthesis of H_2_S in mammals and the observation that small amounts of H_2_S have modulatory effects led to the notion that H_2_S plays a regulatory role by influencing biological processes through its interaction with specific target proteins [152,225].

The cellular production of small amounts of H_2_S has several signaling functions. While high levels of H_2_S are extremely toxic, enzymes in the body can detoxify it by oxidation to harmless sulfate.

H_2_S has attracted considerable interest as a potential pharmacological target and as an important endogenous gas mediator. Drugs that can release H_2_S slowly are being designed to act beneficially on a variety of diseases. The efficacy, safety and mechanism of action are not fully understood.

Several research papers have addressed the therapeutic properties of externally administered H_2_S, suggesting that the use of H2S donor medications may be beneficial for specific pathological conditions. For example, the administration of exogenous H_2_S drugs was found to improve blood vessel function in animal models of diabetes, suggesting a potential therapeutic benefit for diabetic patients. It has been observed that exogenous H_2_S administration promotes angiogenesis, the process of blood vessel formation. This finding suggests that H_2_S may be a potential therapeutic intervention in chronic ischemic diseases.

In further research, it is essential to comprehensively evaluate other biological functions of garlic while isolating and identifying the specific chemicals contained in garlic. Further research should be conducted to further explore the underlying mechanisms by which garlic exerts its effects. In addition, more clinical studies should be carried out to confirm the positive effects of garlic on human health. The risks and negative consequences of garlic consumption should be emphasized.

## Figures and Tables

**Table 1:** Rate constants determined for reactions of H_2_O_2_, ONOOH and HOCl with H_2_S.

Oxidizing	k_2_ M^−1^ s^−1^	pH	Temperature	Reference
Hydrogen peroxide	0.73	7.4	37 °C	[149]
Peroxynitrous acid	6.7 × 10^3^	7.4	37 °C	[150]
Hypochlorous acid	0.8–20 × 10^8^	7.4		[151]
Taurine chloramine	3 × 10^2^	7.4	37 °C	[152]
